# *Unraveling Amazon tree community assembly using Maximum Information Entropy*: a quantitative analysis of tropical forest ecology

**DOI:** 10.1038/s41598-023-28132-y

**Published:** 2023-02-17

**Authors:** Edwin Pos, Luiz de Souza Coelho, Diogenes de Andrade Lima Filho, Rafael P. Salomão, Iêda Leão Amaral, Francisca Dionízia de Almeida Matos, Carolina V. Castilho, Oliver L. Phillips, Juan Ernesto Guevara, Marcelo de Jesus Veiga Carim, Dairon Cárdenas López, William E. Magnusson, Florian Wittmann, Mariana Victória Irume, Maria Pires Martins, Daniel Sabatier, José Renan da Silva Guimarães, Jean-François Molino, Olaf S. Bánki, Maria Teresa Fernandez Piedade, Nigel C. A. Pitman, Abel Monteagudo Mendoza, José Ferreira Ramos, Joseph E. Hawes, Everton José Almeida, Luciane Ferreira Barbosa, Larissa Cavalheiro, Márcia Cléia Vilela dos Santos, Bruno Garcia Luize, Evlyn Márcia Moraes de Leão Novo, Percy Núñez Vargas, Thiago Sanna Freire Silva, Eduardo Martins Venticinque, Angelo Gilberto Manzatto, Neidiane Farias Costa Reis, John Terborgh, Katia Regina Casula, Euridice N. Honorio Coronado, Juan Carlos Montero, Beatriz S. Marimon, Ben Hur Marimon-Junior, Ted R. Feldpausch, Alvaro Duque, Chris Baraloto, Nicolás Castaño Arboleda, Julien Engel, Pascal Petronelli, Charles Eugene Zartman, Timothy J. Killeen, Rodolfo Vasquez, Bonifacio Mostacedo, Rafael L. Assis, Jochen Schöngart, Hernán Castellanos, Marcelo Brilhante de Medeiros, Marcelo Fragomeni Simon, Ana Andrade, José Luís Camargo, Layon O. Demarchi, William F. Laurance, Susan G. W. Laurance, Emanuelle de Sousa Farias, Maria Aparecida Lopes, José Leonardo Lima Magalhães, Henrique Eduardo Mendonça Nascimento, Helder Lima de Queiroz, Gerardo A. C. Aymard, Roel Brienen, Juan David Cardenas Revilla, Flávia R. C. Costa, Adriano Quaresma, Ima Célia Guimarães Vieira, Bruno Barçante Ladvocat Cintra, Pablo R. Stevenson, Yuri Oliveira Feitosa, Joost F. Duivenvoorden, Hugo F. Mogollón, Leandro Valle Ferreira, James A. Comiskey, Freddie Draper, José Julio de Toledo, Gabriel Damasco, Nállarett Dávila, Roosevelt García-Villacorta, Aline Lopes, Alberto Vicentini, Janaína Costa Noronha, Flávia Rodrigues Barbosa, Rainiellen de Sá Carpanedo, Thaise Emilio, Carolina Levis, Domingos de Jesus Rodrigues, Juliana Schietti, Priscila Souza, Alfonso Alonso, Francisco Dallmeier, Vitor H. F. Gomes, Jon Lloyd, David Neill, Daniel Praia Portela de Aguiar, Alejandro Araujo-Murakami, Luzmila Arroyo, Fernanda Antunes Carvalho, Fernanda Coelho de Souza, Dário Dantas do Amaral, Kenneth J. Feeley, Rogerio Gribel, Marcelo Petratti Pansonato, Jos Barlow, Erika Berenguer, Joice Ferreira, Paul V. A. Fine, Marcelino Carneiro Guedes, Eliana M. Jimenez, Juan Carlos Licona, Maria Cristina Peñuela Mora, Carlos A. Peres, Boris Eduardo Villa Zegarra, Carlos Cerón, Terry W. Henkel, Paul Maas, Marcos Silveira, Juliana Stropp, Raquel Thomas-Caesar, Tim R. Baker, Doug Daly, Kyle G. Dexter, John Ethan Householder, Isau Huamantupa-Chuquimaco, Toby Pennington, Marcos Ríos Paredes, Alfredo Fuentes, José Luis Marcelo Pena, Miles R. Silman, J. Sebastián Tello, Jerome Chave, Fernando Cornejo Valverde, Anthony Di Fiore, Renato Richard Hilário, Juan Fernando Phillips, Gonzalo Rivas-Torres, Tinde R. van Andel, Patricio von Hildebrand, Edelcilio Marques Barbosa, Luiz Carlos de Matos Bonates, Hilda Paulette Dávila Doza, Émile Fonty, Ricardo Zárate Gómez, Therany Gonzales, George Pepe Gallardo Gonzales, Jean-Louis Guillaumet, Bruce Hoffman, André Braga Junqueira, Yadvinder Malhi, Ires Paula de Andrade Miranda, Linder Felipe Mozombite Pinto, Adriana Prieto, Agustín Rudas, Ademir R. Ruschel, Natalino Silva, César I. A. Vela, Vincent Antoine Vos, Egleé L. Zent, Stanford Zent, Bianca Weiss Albuquerque, Angela Cano, Diego F. Correa, Janaina Barbosa Pedrosa Costa, Bernardo Monteiro Flores, Milena Holmgren, Marcelo Trindade Nascimento, Alexandre A. Oliveira, Hirma Ramirez-Angulo, Maira Rocha, Veridiana Vizoni Scudeller, Rodrigo Sierra, Milton Tirado, Maria Natalia Umaña, Geertje van der Heijden, Emilio Vilanova Torre, Corine Vriesendorp, Ophelia Wang, Kenneth R. Young, Manuel Augusto Ahuite Reategui, Cláudia Baider, Henrik Balslev, Sasha Cárdenas, Luisa Fernanda Casas, William Farfan-Rios, Cid Ferreira, Reynaldo Linares-Palomino, Casimiro Mendoza, Italo Mesones, Armando Torres-Lezama, Ligia Estela Urrego Giraldo, Daniel Villarroel, Roderick Zagt, Miguel N. Alexiades, Karina Garcia-Cabrera, Lionel Hernandez, William Milliken, Walter Palacios Cuenca, Susamar Pansini, Daniela Pauletto, Freddy Ramirez Arevalo, Adeilza Felipe Sampaio, Elvis H. Valderrama Sandoval, Luis Valenzuela Gamarra, Gerhard Boenisch, Jens Kattge, Nathan Kraft, Aurora Levesley, Karina Melgaço, Georgia Pickavance, Lourens Poorter, Hans ter Steege

**Affiliations:** 1 Quantitative Biodiversity Dynamics, Ecology and Biodiversity, Utrecht University Botanic Gardens, Utrecht University, Padualaan 8, Utrecht, 3584 CH The Netherlands; 2 Naturalis Biodiversity Center, PO Box 9517, Leiden, 2300 RA The Netherlands; 3 Coordenação de Biodiversidade, Instituto Nacional de Pesquisas da Amazônia - INPA, Av. André Araújo, 2936, Petrópolis, Manaus, AM 69067-375 Brazil; 4 Programa Professor Visitante Nacional Sênior Na Amazônia - CAPES, Universidade Federal Rural da Amazônia, Av. Perimetral, s/n, Belém, PA Brazil; 5 Coordenação de Botânica, Museu Paraense Emílio Goeldi, Av. Magalhães Barata 376, C.P. 399, Belém, PA 66040-170 Brazil; 6 EMBRAPA – Centro de Pesquisa Agroflorestal de Roraima, BR 174, km 8 – Distrito Industrial, Boa Vista, RR 69301-970 Brazil; 7 School of Geography, University of Leeds, Woodhouse Lane, Leeds, LS2 9JT UK; 8 Grupo de Investigación en Biodiversidad, Medio Ambiente y Salud-BIOMAS, Universidad de Las Américas, Campus Queri, Quito, Ecuador; 9 Keller Science Action Center, The Field Museum, 1400 S. Lake Shore Drive, Chicago, IL 60605-2496 USA; 10 Departamento de Botânica, Instituto de Pesquisas Científicas e Tecnológicas do Amapá - IEPA, Rodovia JK, Km 10, Campus Do IEPA da Fazendinha, Amapá, 68901-025 Brazil; 11 Herbario Amazónico Colombiano, Instituto SINCHI, Calle 20 No 5-44, Bogotá, DC Colombia; 12 Coordenação de Pesquisas em Ecologia, Instituto Nacional de Pesquisas da Amazônia - INPA, Av. André Araújo, 2936, Petrópolis, Manaus, AM 69067-375 Brazil; 13 Department of Wetland Ecology, Institute of Geography and Geoecology, Karlsruhe Institute of Technology - KIT, Josefstr.1, 76437 Rastatt, Germany; 14 Biogeochemistry, Max Planck Institute for Chemistry, Hahn-Meitner Weg 1, 55128 Mainz, Germany; 15 AMAP, IRD, Cirad, CNRS, INRA, Université de Montpellier, 34398 Montpellier, France; 16 Coordenação de Dinâmica Ambiental, Instituto Nacional de Pesquisas da Amazônia - INPA, Av. André Araújo, 2936, Petrópolis, Manaus, AM 69067-375 Brazil; 17 Science and Education, The Field Museum, 1400 S. Lake Shore Drive, Chicago, IL 60605-2496 USA; 18 Jardín Botánico de Missouri, Oxapampa, Pasco, Peru; 19 Applied Ecology Research Group, School of Life Sciences, Anglia Ruskin University, East Road, Cambridge, CB1 1PT UK; 20 ICNHS, Universidade Federal de Mato Grosso, Av. Alexandre Ferronato, 1200, Sinop, MT 78557-267 Brazil; 21 Departamento de Ecologia, Universidade Estadual Paulista - UNESP – Instituto de Biociências – IB, Av. 24 A, 1515, Bela Vista, Rio Claro, SP 13506-900 Brazil; 22 Divisao de Sensoriamento Remoto – DSR, Instituto Nacional de Pesquisas Espaciais – INPE, Av. Dos Astronautas, 1758, Jardim da Granja, São José Dos Campos, SP 12227-010 Brazil; 23 Herbario Vargas, Universidad Nacional de San Antonio Abad del Cusco, Avenida de La Cultura, Nro 733, Cusco, Cuzco, Peru; 24 Biological and Environmental Sciences, University of Stirling, Stirling, FK9 4LA UK; 25 Centro de Biociências, Departamento de Ecologia, Universidade Federal do Rio Grande do Norte, Av. Senador Salgado Filho, 3000, Natal, RN 59072-970 Brazil; 26 Departamento de Biologia, Universidade Federal de Rondônia, Rodovia BR 364 s/n Km 9, 5 - Sentido Acre, Unir, Porto Velho, RO 76.824-027 Brazil; 27 Programa de Pós- Graduação em Biodiversidade e Biotecnologia PPG- Bionorte, Universidade Federal de Rondônia, Campus Porto Velho Km 9, 5 Bairro Rural, Porto Velho, RO 76.824-027 Brazil; 28 Department of Biology and Florida Museum of Natural History, University of Florida, Gainesville, FL 32611 USA; 29 Centre for Tropical Environmental and Sustainability Science and College of Science and Engineering, James Cook University, Cairns, QLD 4870 Australia; 30 Instituto de Investigaciones de la Amazonía Peruana (IIAP), Av. A. Quiñones Km 2,5, Iquitos, Loreto, 784 Peru; 31 Instituto Boliviano de Investigacion Forestal, Av. 6 de Agosto #28, Km. 14, Doble via La Guardia, 6204 Santa Cruz, Santa Cruz, Casilla Bolivia; 32 Programa de Pós-Graduação em Ecologia e Conservação, Universidade do Estado de Mato Grosso, Nova Xavantina, MT Brazil; 33 Geography, College of Life and Environmental Sciences, University of Exeter, Rennes Drive, Exeter, EX4 4RJ UK; 34 Departamento de Ciencias Forestales, Universidad Nacional de Colombia, Calle 64 X Cra 65, 1027 Medellín, Antioquia Colombia; 35 International Center for Tropical Botany (ICTB) Department of Biological Sciences, Florida International University, 11200 SW 8Th Street, OE 243, Miami, FL 33199 USA; 36 Cirad UMR Ecofog, AgrosParisTech, CNRS, INRA, Univ Guyane, Campus Agronomique, 97379 Kourou Cedex, France; 37 Agteca-Amazonica, Santa Cruz, Bolivia; 38 Facultad de Ciencias Agrícolas, Universidad Autónoma Gabriel René Moreno, Santa Cruz, Santa Cruz, Bolivia; 39 Natural History Museum, University of Oslo, Postboks 1172, 0318 Oslo, Norway; 40 Centro de Investigaciones Ecológicas de Guayana, Universidad Nacional Experimental de Guayana, Calle Chile, Urbaniz Chilemex, Puerto Ordaz, Bolivar, Venezuela; 41 Prédio da Botânica e Ecologia, Embrapa Recursos Genéticos e Biotecnologia, Parque Estação Biológica, Av. W5 Norte, Brasilia, DF 70770-917 Brazil; 42 Projeto Dinâmica Biológica de Fragmentos Florestais, Instituto Nacional de Pesquisas da Amazônia - INPA, Av. André Araújo 2936, Petrópolis, Manaus, AM 69067-375 Brazil; 43 Laboratório de Ecologia de Doenças Transmissíveis da Amazônia (EDTA), Instituto Leônidas e Maria Deane, Fiocruz, Rua Terezina, 476, Adrianópolis, Manaus, AM 69060-001 Brazil; 44 Programa de Pós-Graduação em Biodiversidade e Saúde, Instituto Oswaldo Cruz - IOC/FIOCRUZ, Pav. Arthur Neiva – Térreo, Av. Brasil, 4365 – Manguinhos, Rio de Janeiro, RJ 21040-360 Brazil; 45 Instituto de Ciências Biológicas, Universidade Federal do Pará, Av. Augusto Corrêa 01, Belém, PA 66075-110 Brazil; 46 Programa de Pós-Graduação em Ecologia, Universidade Federal do Pará, Av. Augusto Corrêa 01, Belém, PA 66075-110 Brazil; 47 Embrapa Amazônia Oriental, Trav. Dr. Enéas Pinheiro S/nº, Belém, PA 66095-100 Brazil; 48 Diretoria Técnico-Científica, Instituto de Desenvolvimento Sustentável Mamirauá, Estrada do Bexiga, 2584, Tefé, AM 69470-000 Brazil; 49 Programa de Ciencias del Agro y el Mar, Herbario Universitario (PORT), UNELLEZ-Guanare, Guanare, Portuguesa, 3350 Venezuela; 50 Instituto de Biociências – Department of Botanica, Universidade de Sao Paulo - USP, Rua do Matão 277, Cidade Universitária, São Paulo, SP 05508-090 Brazil; 51 Laboratorio de Ecología de Bosques Tropicales y Primatología, Universidad de los Andes, Carrera 1 # 18a- 10, 111711 Bogotá, DC Colombia; 52 Programa de Pós-Graduação Em Biologia (Botânica), Instituto Nacional de Pesquisas da Amazônia - INPA, Av. André Araújo, 2936, Petrópolis, Manaus, AM 69067-375 Brazil; 53 Institute of Biodiversity and Ecosystem Dynamics, University of Amsterdam, Sciencepark 904, Amsterdam, 1098 XH The Netherlands; 54 Endangered Species Coalition, 8530 Geren Rd., Silver Spring, MD 20901 USA; 55 Inventory and Monitoring Program, National Park Service, 120 Chatham Lane, Fredericksburg, VA 22405 USA; 56 Center for Conservation and Sustainability, Smithsonian Conservation Biology Institute, 1100 Jefferson Dr. SW, Suite 3123, Washington, DC 20560-0705 USA; 57 Department of Global Ecology, Carnegie Institution for Science, 260 Panama St., Stanford, CA 94305 USA; 58 Universidade Federal do Amapá, Ciências Ambientais, Rod. Juscelino Kubitschek km2, Macapá, AP 68902-280 Brazil; 59 Department of Integrative Biology, University of California, Berkeley, CA 94720-3140 USA; 60 Biologia Vegetal, Universidade Estadual de Campinas, Caixa Postal 6109, Campinas, SP 13.083-970 Brazil; 61 Department of Ecology and Evolutionary Biology, Cornell University, Corson Hall, 215 Tower Road, Ithaca, NY 14850 USA; 62 Peruvian Center for Biodiversity and Conservation (PCBC), Iquitos, Peru; 63 Department of Ecology, University of Brasilia, Brasilia, DF 70904-970 Brazil; 64 ICNHS, Federal University of Mato Grosso, Av. Alexandre Ferronato 1200, Setor Industrial, Sinop, MT 78.557-267 Brazil; 65 Natural Capital and Plant Health, Royal Botanic Gardens, Kew, Richmond, TW9 3AB Surrey UK; 66 Programa de Pós-Graduação em Ecologia, Instituto Nacional de Pesquisas da Amazônia - INPA, Av. André Araújo, 2936, Petrópolis, Manaus, AM 69067-375 Brazil; 67 Forest Ecology and Forest Management Group, Wageningen University and Research, Droevendaalsesteeg 3, P.O. Box 47, Wageningen, 6700 AA The Netherlands; 68 Escola de Negócios Tecnologia e Inovação, Centro Universitário do Pará, Belém, PA Brazil; 69 Universidade Federal do Pará, Rua Augusto Corrêa 01, Belém, PA 66075-110 Brazil; 70 Faculty of Natural Sciences, Department of Life Sciences, Imperial College London, South Kensington Campus, Silwood ParkLondon, SW7 2AZ UK; 71 Ecosistemas, Biodiversidad y Conservación de Especies, Universidad Estatal Amazónica, Km. 2 1/2 Vía a Tena (Paso Lateral), Puyo, Pastaza, Ecuador; 72 Museo de Historia Natural Noel Kempff Mercado, Universidad Autónoma Gabriel Rene Moreno, Avenida Irala 565 Casilla Post Al 2489, Santa Cruz, Santa Cruz, Bolivia; 73 Departamento de Genética, Ecologia e Evolução, Universidade Federal de Minas Gerais, Instituto de Ciências Biológicas, Av. Antônio Carlos, 6627 Pampulha, Belo Horizonte, MG 31270-901 Brazil; 74 Department of Biology, University of Miami, Coral Gables, FL 33146 USA; 75 Fairchild Tropical Botanic Garden, Coral Gables, FL 33156 USA; 76 Instituto de Biociências - Dept. Ecologia, Universidade de Sao Paulo - USP, Rua do Matão, Trav. 14, No. 321, Cidade Universitária, São Paulo, SP 05508-090 Brazil; 77 Lancaster Environment Centre, Lancaster University, Lancaster, LA1 4YQ Lancashire UK; 78 Environmental Change Institute, University of Oxford, Oxford, OX1 3QY Oxfordshire UK; 79 Empresa Brasileira de Pesquisa Agropecuária, Embrapa Amapá, Rod. Juscelino Kubitschek Km 5, Macapá, Amapá 68903-419 Brazil; 80 Grupo de Ecología y Conservación de Fauna y Flora Silvestre, Instituto Amazónico de Investigaciones Imani, Universidad Nacional de Colombia Sede Amazonia, Leticia, Amazonas Colombia; 81 Universidad Regional Amazónica IKIAM, Km 7 Via Muyuna, Tena Napo, Ecuador; 82 School of Environmental Sciences, University of East Anglia, Norwich, NR4 7TJ UK; 83 Direccíon de Evaluación Forestal y de Fauna Silvestre, Av. Javier Praod Oeste 693, Magdalena del Mar, Peru; 84 Escuela de Biología Herbario Alfredo Paredes, Universidad Central, Ap. Postal 17.01.2177, Quito, Pichincha Ecuador; 85 Department of Biological Sciences, Humboldt State University, 1 Harpst Street, Arcata, CA 95521 USA; 86 Museu Universitário / Centro de Ciências Biológicas e da Natureza / Laboratório de Botânica e Ecologia Vegetal, Universidade Federal do Acre, Rio Branco, AC 69915-559 Brazil; 87 Institute of Biological and Health Sciences, Federal University of Alagoas, Av. Lourival Melo Mota, s/n, Tabuleiro do Martins, Maceio, AL 57072-970 Brazil; 88 Iwokrama International Centre for Rain Forest Conservation and Development, Georgetown, Guyana; 89 New York Botanical Garden, 2900 Southern Blvd, Bronx, New York, NY 10458-5126 USA; 90 School of Geosciences, University of Edinburgh, 201 Crew Building, King’s Buildings, Edinburgh, EH9 3JN UK; 91 Tropical Diversity Section, Royal Botanic Garden Edinburgh, 20a Inverleith Row, Edinburgh, EH3 5LR Scotland, UK; 92 Servicios de Biodiversidad EIRL, Jr. Independencia 405, Iquitos, Loreto, 784 Peru; 93 Herbario Nacional de Bolivia, Universitario UMSA, Casilla 10077 Correo Central, La Paz, La Paz, Bolivia; 94 Center for Conservation and Sustainable Development, Missouri Botanical Garden, P.O. Box 299, St. Louis, MO 63166-0299 USA; 95 Universidad Nacional de Jaén, Carretera Jaén San Ignacio Km 23, Jaén, Cajamarca, 06801 Peru; 96 Biology Department and Center for Energy, Environment and Sustainability, Wake Forest University, 1834 Wake Forest Rd, Winston Salem, NC 27106 USA; 97 Laboratoire Evolution et Diversité Biologique, CNRS and Université Paul Sabatier, UMR 5174 EDB, 31000 Toulouse, France; 98 Andes to Amazon Biodiversity Program, Madre de Dios, Madre de Dios, Peru; 99 Department of Anthropology, University of Texas at Austin, SAC 5.150, 2201 Speedway Stop C3200, Austin, TX 78712 USA; 100 Fundación Puerto Rastrojo, Cra 10 No. 24-76 Oficina 1201, Bogotá, DC Colombia; 101 Colegio de Ciencias Biológicas y Ambientales-COCIBA and Galapagos Institute for the Arts and Sciences-GAIAS, Universidad San Francisco de Quito-USFQ, Quito, Pichincha Ecuador; 102 Department of Wildlife Ecology and Conservation, University of Florida, 110 Newins-Ziegler Hall, Gainesville, FL 32611 USA; 103 Biosystematics Group, Wageningen University, Droevendaalsesteeg 1, Wageningen, 6708 PB The Netherlands; 104 Fundación Estación de Biología, Cra 10 No. 24-76 Oficina, 1201 Bogotá, DC Colombia; 105 Direction Régionale de la Guyane, ONF, Cayenne, 97300 French Guiana; 106 PROTERRA, Instituto de Investigaciones de la Amazonía Peruana (IIAP), Av. A. Quiñones Km 2,5, Iquitos, Loreto, 784 Peru; 107 ACEER Foundation, Jirón Cusco N° 370, Puerto Maldonado, Madre de Dios, Peru; 108 Departement EV, Muséum National d’histoire Naturelle de Paris, 16 Rue Buffon, Paris, 75005 France; 109 Amazon Conservation Team, Doekhieweg Oost #24, Paramaribo, Suriname; 110 Institut de Ciència i Tecnologia Ambientals, Universitat Autònoma de Barcelona, 08193 Bellaterra, Barcelona, Spain; 111 Environmental Change Institute, Dyson Perrins Building, Oxford University Centre for the Environment, South Parks Road, Oxford, OX1 3QY England, UK; 112 Instituto de Ciencias Naturales, Universidad Nacional de Colombia, 7945 Apartado, Bogotá, DC Colombia; 113 Instituto de Ciência Agrárias, Universidade Federal Rural da Amazônia, Av. Presidente Tancredo Neves 2501, Belém, PA 66.077-830 Brazil; 114 Escuela Profesional de Ingeniería Forestal, Universidad Nacional de San Antonio Abad del Cusco, Jirón San Martín 451, Puerto Maldonado, Madre de Dios, Peru; 115 Universidad Autónoma del Beni José Ballivián, Campus Universitario Final, Av. Ejercito, Riberalta, Beni, Bolivia; 116 Laboratory of Human Ecology, Instituto Venezolano de Investigaciones Científicas - IVIC, Ado 20632, Caracas, 1020A DC Venezuela; 117 Cambridge University Botanic Garden, 1 Brookside., Cambridge, CB2 1JE UK; 118 School of Agriculture and Food Sciences - ARC Centre of Excellence for Environmental Decisions CEED, The University of Queensland, St. Lucia, QLD 4072 Australia; 119 Plant Biology Department, Rua Monteiro Lobato, University of Campinas, 255, Cidade Universitária Zeferino Vaz, Barão Geraldo, Campinas, São Paulo, CEP 13083-862 Brazil; 120 Resource Ecology Group, Wageningen University and Research, Droevendaalsesteeg 3a, Lumen, Building Number 100, Wageningen, Gelderland 6708 PB The Netherlands; 121 Laboratório de Ciências Ambientais, Universidade Estadual do Norte Fluminense, Av. Alberto Lamego 2000, Campos dos, Goyatacazes, RJ 28013-620 Brazil; 122 Instituto de Investigaciones Para el Desarrollo Forestal (INDEFOR), Universidad de los Andes, Conjunto Forestal, Mérida, Mérida, 5101 Venezuela; 123 Departamento de Biologia, Universidade Federal do Amazonas - UFAM – Instituto de Ciências Biológicas – ICB1, Av General Rodrigo Octavio 6200, Manaus, AM 69080-900 Brazil; 124 GeoIS, el Día 369 y el Telégrafo, 3° Piso, Quito, Pichincha Ecuador; 125 Department of Ecology and Evolutionary Biology, University of Michigan, Ann Arbor, MI 48109 USA; 126 University of Nottingham, University Park, Nottingham, NG7 2RD UK; 127 School of Environmental and Forest Sciences, University of Washington, Seattle, WA 98195-2100 USA; 128 Environmental Science and Policy, Northern Arizona University, Flagstaff, AZ 86011 USA; 129 Geography and the Environment, University of Texas at Austin, 305 E. 23Rd Street, CLA Building, Austin, TX 78712 USA; 130 Medio Ambiente, PLUSPRETOL, Iquitos, Loreto, Peru; 131 The Mauritius Herbarium, Agricultural Services, Ministry of Agro-Industry and Food Security, Reduit, 80835 Mauritius; 132 Department of Bioscience, Aarhus University, Building 1540 Ny Munkegade, 8000 Aarhus C, Aarhus, Denmark; 133 Living Earth Collaborative, Washington University in Saint Louis, St. Louis, MO 63130 USA; 134 Escuela de Ciencias Forestales (ESFOR), Universidad Mayor de San Simon (UMSS), Sacta, Cochabamba, Bolivia; 135 FOMABO, Manejo Forestal en Las Tierras Tropicales de Bolivia, Sacta, Cochabamba, Bolivia; 136 Tropenbos International, Lawickse Allee 11, PO Box 232, Wageningen, 6700 AE The Netherlands; 137 School of Anthropology and Conservation, University of Kent, Marlowe Building, Canterbury, Kent, CT2 7NR UK; 138 Herbario Nacional del Ecuador, Universidad Técnica del Norte, Quito, Pichincha Ecuador; 139 Instituto de Biodiversidade e Floresta, Universidade Federal do Oeste do Pará, Rua Vera Paz, Campus Tapajós, Santarém, PA 68015-110 Brazil; 140 Facultad de Biologia, Universidad Nacional de la Amazonia Peruana, Pevas 5Ta Cdra, Iquitos, Loreto, Peru; 141 Department of Biology, University of Missouri, St. Louis, MO 63121 USA; 142 Department of Biogeochemical Integration, Max-Planck-Institute for Biogeochemistry, P.O. Box 10 01 64, 07701 Jena, Germany; 143 Functional Biogeography, Max-Planck-Institute for Biogeochemistry, P.O. Box 10 01 64, 07701 Jena, Germany; 144 Department of Ecology and Evolutionary Biology, UCLA, 621 Charles E. Young Drive South, Box 951606, Los Angeles, CA 90095 USA

**Keywords:** Computational biology and bioinformatics, Ecology, Statistical physics, thermodynamics and nonlinear dynamics

## Abstract

In a time of rapid global change, the question of what determines patterns in species abundance distribution remains a priority for understanding the complex dynamics of ecosystems. The constrained maximization of information entropy provides a framework for the understanding of such complex systems dynamics by a quantitative analysis of important constraints via predictions using least biased probability distributions. We apply it to over two thousand hectares of Amazonian tree inventories across seven forest types and thirteen functional traits, representing major global axes of plant strategies. Results show that constraints formed by regional relative abundances of genera explain eight times more of local relative abundances than constraints based on directional selection for specific functional traits, although the latter does show clear signals of environmental dependency. These results provide a quantitative insight by inference from large-scale data using cross-disciplinary methods, furthering our understanding of ecological dynamics.

## Introduction

**Figure 1:**
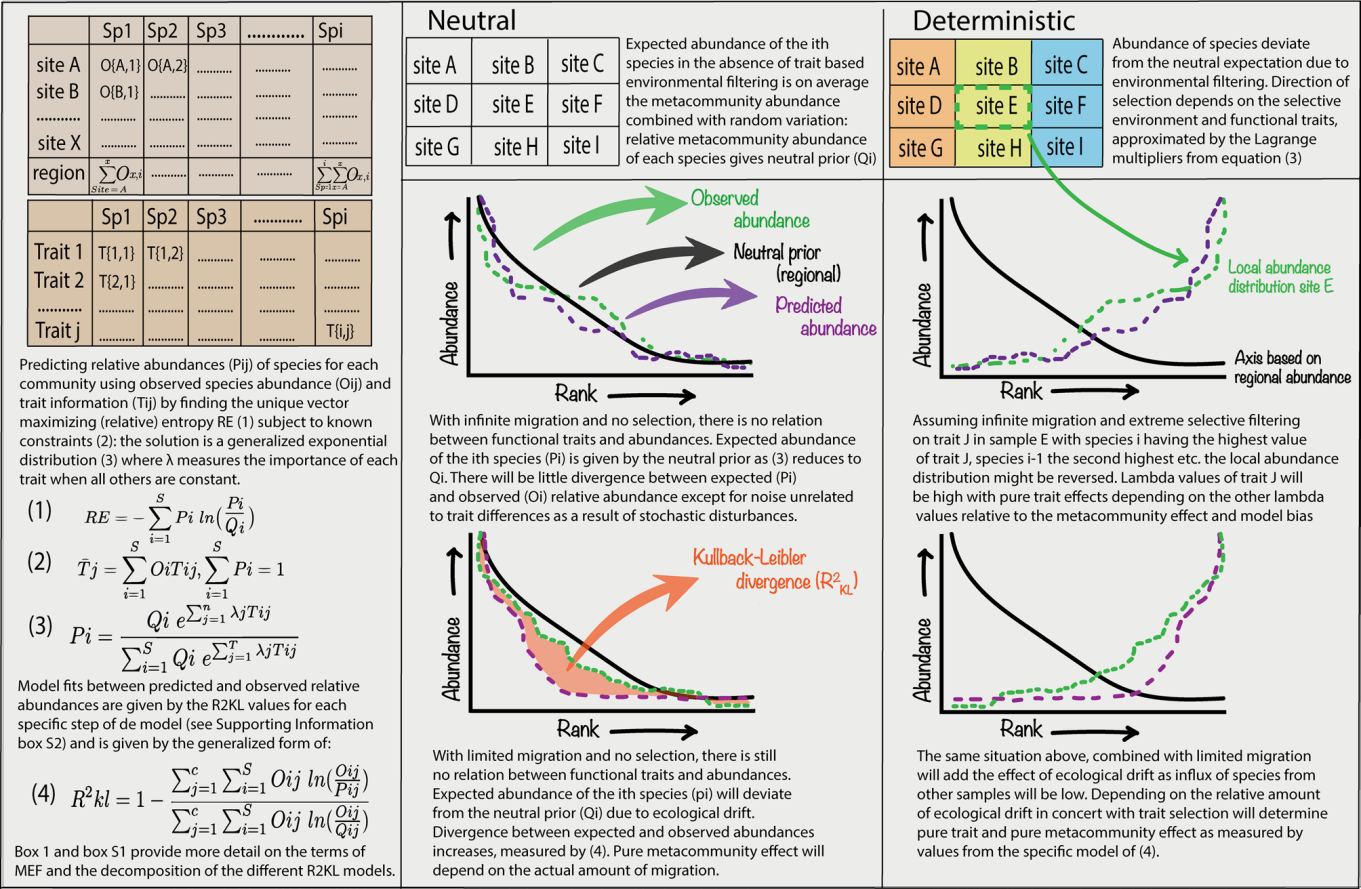
Schematic depiction of the MEF procedure. Left panel shows a genus abundances per site and a functional trait matrix per genus, bottom half outlines calculations. Middle and right panel show different scenarios of neutral and deterministic dynamics under infinite or limited migration. Figure was custom made using Adobe Illustrator (Adobe Inc., 2019. Adobe Illustrator).

Drivers of species distributions and their predictions have been a long-standing search in ecology, with approaches varying from deterministic to neutral (i.e. stochastic) and almost everything in between (e.g. near-neutral, continuum or emergent-neutral^1,2^). Most models are based on prior assumptions of processes that drive community dynamics. The Maximum Entropy Formalism (hereafter called MEF) makes no such, potentially unjustified, a-priori assumptions in generating predictions of species abundance distributions, as such it is a useful construct to infer processes driving community dynamics given the constraints imposed by prior knowledge (e.g. functional traits or summed regional abundances)^3^. Quantifying the relative importance of these distinct constraints can thus provide additional answers to understand the complexity of community dynamics (see Supporting Materials SM: boxes S1–S3). This is especially so because, although many different tests are available that link variation in taxon abundances to (1) trait variation, (2) taxon turnover between habitats or environments and (3) the distance decay of similarities between samples, none quantify the importance of these relative to each other. The MEF as applied here, however, is capable of and designed to do exactly this by decomposing variation to separate information explained by each of these aspects in a four-step model (Fig. 1 and Box S2). Its application to an unprecedented large tree inventory database on genus level taxonomy consisting of > 2,000 1-ha plots distributed over Amazonia^4^ and a genus trait database of 13 key functional traits representing global axes of plant strategies^5^ allows us to advance the study of Amazonian tree community dynamics from a new cross-disciplinary perspective.

## Results

Principles from information theory^6–8^ can be used in an ecological setting to predict the most likely abundance state for each taxon while simultaneously maximizing entropy based on constraints. Maximization of entropy allows quantifying the information yield for each constraint and therefor identifies which constraints reduce entropy the most. Here we specifically use Shipley’s mathematical framework (CATS) for the MEF calculations, similar to earlier studies^9–11^.

### Predictive power of the four-step model

**Figure 2:**
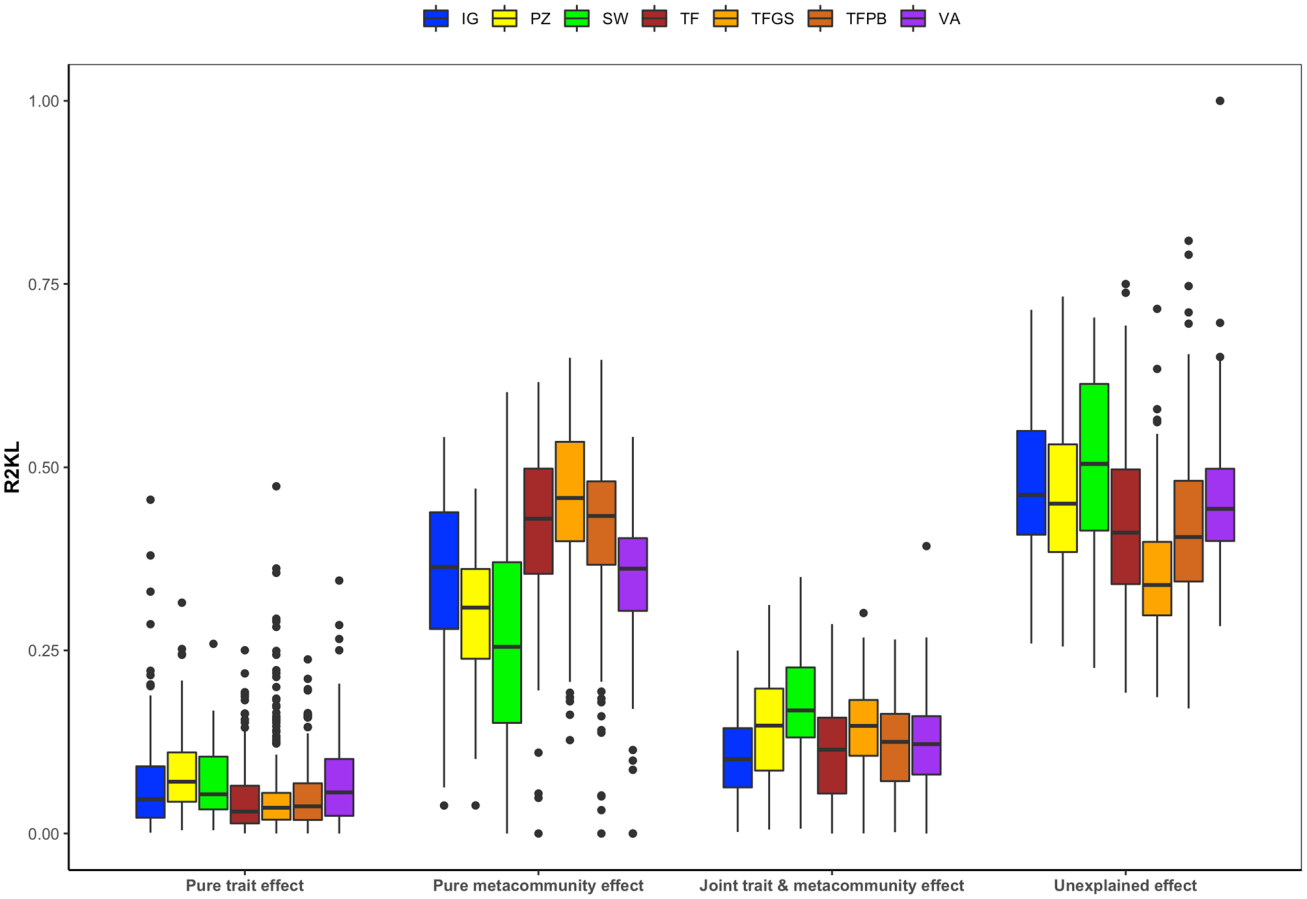
Visual representation of pure trait, pure metacommunity, hybrid model and the remaining unexplained information for each separate forest type. Abbreviations indicate different types: igapó (IG), podzol (PZ), swamp (SW), Brazilian shield terra firme (TFBS), Guiana Shield terra firme (TFGS), Pebas terra firme (TFPB) and várzea (VA). Boxplots show median value of pure effects over all samples, with lower and upper hinges corresponding to 25th and 75th percentiles. Whiskers extends from hinge to largest or smallest value no further than 1.5 * IQR from hinge. Points beyond this range are plotted individually and only positive values were plotted.

Using a uniform prior and CWM values (Community Weighted Means) as constraints accounted for 23% on average of total deviance between observed and predicted relative abundances (measured by R^2^_KL_ values, see Box S2 Eq. 5). Filtered by forest type this was 34% for podzol forests, *várzea* 25%, *igapó* 23%, swamp forests 34%, 21% and 24% for Guyana Shield and Pebas *terra firme* respectively and 20% for Brazilian Shield *terra firme* forests (see Table S1 for detailed decomposition). Using observed metacommunity relative abundances as prior regardless of functional traits accounted for on average 56% for the combined dataset with for all forest types between 51 and 55%, except for the Guyana Shield *terra firme* with 62%. The hybrid model (including both traits as constraints and the metacommunity prior) performed slightly better for the combined dataset (average 60%) with a minimum of 57% for swamp and *várzea* forests and a maximum of 66% for Guyana Shield *terra firme* forests. To compensate for spurious relationships between regional abundances and local trait constraints, regardless of selection, explanatory power was regarded relative to model bias yielding the pure trait and metacommunity effects (Box S3, Fig. 2 and Table S1). This lowered the proportion of information accounted for and yielded average pure metacommunity effects of 40% for the overall dataset ranging between 26 and 45% for each forest type separately with pure trait effects explaining only 5% of information for the combined dataset on average with for each forest type between 3 and 9%. Although the latter was lowered substantially, the explanatory power did appear to be strongly dependent on forest type. The online supplementary material provides additional results relating to the predictive power of each model as well as the spatial gradient of the pure trait and metacommunity effects (Figs. S2–S3).

### Direction and strength of selection of trait-based constraints

Each trait showed significant differences in lambda when compared between forest types (Fig. S1, see methods for a definition of lambda). Scatterplots of CWM trait values versus lambda show that, in general, higher lambda values correspond with higher CWM trait values (Fig. S7). A number of functional traits associated with low nutrient conditions (e.g. ectomycorrhiza) and life history strategies suited for protection against herbivores (e.g. latex, resin and high leaf C content) were clearly positively associated with abundance in nutrient poor environments (podzols), indicated by the positive lambda values. In contrast, having fleshy fruits and high leaf N and P content were clearly negatively associated with abundance on these soils. Nodulation was also negatively associated with abundance on poor soils. The ability to accumulate aluminium was positively associated with abundance on those soils commonly associated with higher aluminium content such as *igapó* (strong positive effects). In contrast, it was strongly negatively associated with abundance on other soils, with negative lambda values for podzol and *várzea* forests. Traits such as SLA or winged fruits also showed patterns dependent on forest type, although less pronounced.

### Effect of regional metacommunity prior

There was a remarkable similar mean 22% decrease of the information explained purely by the metacommunity prior for each forest type (Fig. S3). For the separate forest types, although the initial pure metacommunity effect varied, the decline appeared remarkably similar with a mean 25% decrease in pure metacommunity effect for podzol, 23% for *várzea*, 26% for *igapó* and 27% for swamp forests with *terra firme* forests having a smaller decline of approximately 21%, averaged over the three subregions. It should be noted there is an obvious risk that when sampling size is increased, this also includes more environmental heterogeneity as samples are coming from a variety of localities potentially leading to changing composition. If this were the case, however, the regional prior (q_i_ from Fig. 1 and Box S2) would also change, as taxa might be abundant in some places but rare or absent in others. As the metacommunity effect is the explained information that remains relative to any trait effects (i.e. information unique to the neutral prior) and the pure trait effects are the explained information remaining after correcting for pure metacommunity effects (Box S3) this effect should then be accompanied by an increase in pure trait effect for each sample. This was not observed, not even within the different forest types. Instead, the trait effect gradually went up and then remained constant (Fig. S4).

## Discussion

The MEF emerges from a well-founded theoretical and empirical body of ecology and evolutionary biology, regarding natural selection, migration and population dynamics. From an ecological point of view, it can be used to quantify the relative association between directional or stabilizing selection for functional traits versus the importance of relative regional abundance regardless of these traits by imposing these as constraints. Our results show that pure trait effects, on average, explained only 5% of the information when all forest types were taken together whereas the pure metacommunity effect, however, explained eight times more taken all forest types together (40%). Greater trait dissimilarity was positively associated with higher pure trait effects, indicating trait-based selection, although the assumed influence of dispersal regardless of these traits appeared to confer more information explaining tree genus composition of the Amazon rainforest. The strength and direction of selection indicated clear directional selective pressure for life history strategies of either growth or protection, depending on forest type (see supplementary online material S-A for a more detailed exploration of ecological interpretation). Including community weighted variance as reflective of potential stabilizing selection did not provide additional information. Although this could be interpreted as indicative of weak or absent stabilizing selection, it is more likely to be an artefact of many genera not being shared among localities due to the sheer geographical scale resulting in a strong mismatch between observed, predicted and uniform relative abundances resulting in a model bias higher than information yielded by including these constraints (see also box S2).

Despite showing clear patterns in environmental selection and dispersal effects, there was a large proportion of information left unexplained (44% on average). Potentially, local demographic stochasticity could weaken any link between functional traits measured and regional abundances of genera. This would, however, mean that almost half of the information contained in relative abundances are the result of random population dynamics and are not structurally governed. Alternatively, this could be due to functional traits reflective of processes not taken into account in this study, such as traits reflective of interactions between trophic levels (e.g. traits influencing specific plant-pathogen interactions). Another and at least equally likely hypothesis for (local) unexplained information is that when scaling up, the ratio of genus richness to total abundance decreases rapidly initially but stabilizes again as relatively non-overlapping habitats are included in the regional abundance distributions and more genera are included again due to the different habitats. This would result in a change of the regional abundance distribution (i.e. the prior) to which each local community is compared, resulting in higher local unexplained information. Further study into these aspects could provide additional insight, though the data necessary for these scales is lacking for Amazonian trees.

### Metacommunity importance

Although the initial explanatory power of the metacommunity prior differed between forest types, the decay pattern was very similar. As the effects of either traits or the metacommunity are measured in the goodness-of-fit predictions on local relative abundances, this implies that at small spatial scales the surrounding regional abundances provide better estimators than functional traits, while at larger spatial scales this shifts to the traits. The ecological translation would be that on small spatial scales, local communities share similar environmental conditions leaving dispersal and drift acting predominantly in changing community composition, at least for genus level taxonomy. As the potential regional pool is increased, more and more environmental heterogeneity and non-overlapping regions are likely to be introduced. The more gradual decline of *terra firme* forests can then arguably be attributed to these forests having the largest relative surface area of Amazonia (even for the separate subregions), potentially giving these forests an almost continuous metacommunity without gaps, resulting in a more gradual transition from metacommunity to trait relative importance. The fact that metacommunity effects do not change anymore after certain distances would indicate the effect of dispersal potentially occurs over very large distances. It should be noted that as these calculations are done at community and genus level, they do not measure single dispersal events but rather the effect of dispersal on community composition much deeper in time. In other words, this effect suggests more than a dispersal event every now and then. Instead, it argues for prolonged mixing of forests on large geographical and temporal scales, supported by recent findings demonstrating a lack of geographical phylogenetic structure of lineages for Amazonian tree genera^12^.

### Conclusion

Using an unprecedented scale of data and applying the Maximum Entropy Formalism from information theory we show that constraints formed by regional relative abundances of genera explain eight times more of local relative abundances then constraints based on directional selection for specific functional traits, although the latter does show clear signals of environmental dependency. There is, however, still much to be explored due to the large unexplained effects and analyses on finer taxonomic (i.e. species level) and environmental (e.g. microhabitat) scales could resolve these issues. The relatively large effects of the regional pool of genera over great distances does suggest an important role for long term dispersal and mixing of Amazonian trees, especially for the Amazonian interior.

## Methods

### Empirical data

The ATDN^4,13,14^ consists of over 2000 tree inventory plots distributed over the Amazon basin and the Guiana Shield, collectively referred to as Amazonia (a map of all current plots can be found at https://atdn.myspecies.info/). Only those plots with trees ≥ 10 cm diameter at breast height were used, leaving 2011 plots with a mean of 558 individuals per plot identified to at least genus level. Most plots used are 1 ha in size (1414) with 492 being smaller (minimum size of 0.1 ha) and 105 larger (maximum size of 80 ha). Genera have been standardized to the W3 Tropicos database^15^ using the Taxonomic Name Resolution Service (TNRS, see^16^). After filtering based on above criteria and solving nomenclature issues, 1,121,935 individuals belonging to over 828 genera remained. Plots were distributed over seven abiotically different forest types: Podzol forests (PZ), *Igapó* (IG, black water flood forests), *Várzea* (VA white water flood forests), Swamp (SW) and *Terra firme* forests (TF) with subregions BS (Brazilian Shield), GS (Guyana Shield) and PB (Pebas) (see also^17^ for details regarding these forest types). Trait data were extracted from several sources. Wood density was mostly derived from^18^. Traits related to leaf characteristics mostly came from four large datasets^19–24^, including additional data from other sources^25–27^ as well as unpublished data (J. Lloyd, A.A. de Oliveira, L. Poorter, M. van de Sande & Mazzei, M. van de Sande & L. Poorter). Data on seed mass came from^28–30^ as well as different flora’s and tree guides. As this particular trait can vary over several orders of magnitude, this was included on a log-scale^29,31^. Ectomycorrhizal aspects were derived from literature^32^, the same applies to nodulation^33,34^. Traits involved in aluminum accumulation were based on^35,36^ and references therein. For binary traits (yes/no), a genus was considered having a certain trait only when > 50% of the genus was positive for that specific trait.

### Functional traits and trait imputation

Constraints were formed by Community Weighted genus Means (CWM) of functional traits (Table 1), related to key ecological life history aspects. According to principles of natural selection, CWM values will likely be biased towards favourable trait values for that particular environment in the case of directional selection, as taxa with these traits will be more abundant due to environmental selection. Previous studies included community weighted variance (CWV) as well as indicative of potential stabilizing selection^11,37^. In our case, however, including CWV as constraints resulted in a model bias that was consistently higher than information including trait or metacommunity aspects, CWV was therefore not included as constraints in the final analysis. As for many traits it has been shown earlier that the interspecific variability was larger than the intraspecific variability, this allowed the use of data from different sources to at least calculate a mean species trait value. Genus trait values were subsequently computed as genus-level means of species values if known within the genus and considered constant for each genus. Genus level of taxonomy was used as the available trait database had the most information on this taxonomic level (see Table 1). Unknown values for traits were estimated by Multiple Imputation with Chained Equations (MICE, see^15^) by delta adjustment, subtracting a fixed amount (delta), with sensitivity of this adjustment to the imputations of the observed versus imputed data analysed using density plots (Fig. S8) and a linear regression model. This procedure was done using the *mice* package^38^, available on the R repository, under predictive mean matching (*pmm* setting, 50 iterations). Results showed imputations were stable and showed near identical patterns with each imputation scenario (see Figs. S5–S6 and Table S2). After imputation, all trait values were transformed to Community Weighted Means (CWM) of each trait (*J*) for each plot (*K*) (${\overline{T} }_{JK}$) as ${\overline{T} }_{JK}= {\sum }_{i=1}^{S}{t}_{ij}{ra}_{ik}$ with *ra* the relative abundance of the *ith* genus in the *kth* plot following earlier uses^37^.

**Table 1:** Overview of used functional traits.

Functional trait	Units	Mean	SD	Est %	Associated challenge
Wood density (*WD*)	g/cm^3^	0.63	0.17	30	Longevity^43^
Seed mass class (*SMC*)	categorical (1–8)	4.3	1.4	31	Dispersal, Fecundity, Establishment^43^
Specific leaf area (*SLA*)	mm^2^/mg	15	5.9	41	Establishment, Plasticity, Disturbance^43^
Leaf nitrogen content (*N*)	mg/g	22.3	7.30	41	Photosynthetic capacity^43^
Leaf phosphorus content (*P*)	mg/g	1	0.77	50	Limited available P for metabolism^44^
Leaf carbon content (*C*)	mg/g	468	38.1	54	Herbivore resistance (C:N)^45^
Latex	1 = no, 2 = yes	1.2	0.43	46	Herbivore resistance^46^
Resin	1 = no, 2 = yes	1.1	0.35	58	Herbivore resistance^46^
Root nodules (*Nodules*)	1 = no, 2 = yes	1.1	0.28	0	Nitrogen fixation^47^
Ectomycorrhiza (*EctoMyco*)	1 = no, 2 = yes	1.01	0.11	0	Organic N fixation , heavy metal pollution^48^
Aluminum accumulation (*AlAcc*)	1 = no, 2 = yes	1.1	0.21	3	Heavy metal pollution^49^
Fleshy fruits (*Fleshy*)	1 = no, 2 = yes	1.6	0.50	7	Dispersal (*specificity*)^50^
Winged seeds (*Wings*)	1 = no, 2 = yes	1.2	0.42	39	Dispersal (*limitation*)^50^

Mean and standard deviation (SD) are calculated after predictive mean matching (percentage of estimated values is given by Est (%)). Associated challenge indicates different aspects of life history and selective environment related to specific functional traits, sources are given in the footnote. For specific methodology of measurement protocols and calculation for each trait we refer to the original sources of the data (see main text).

### MEF procedure predictions and ecological inference

Figure 1 provides a schematic procedure overview, box S1 provides an overview of important terms and Boxes S2–S3 further mathematical details. Initially, a maximally uninformative prior is specified, where q_i_ (Box S1 Eq. 1) equals 1/S, indicative of each species having equal abundances, and trait constraints are randomly permuted multiple times (n = 50) among genera to test whether inclusion of specified constraints significantly changes derived probability distributions (see also^39^). Subsequently, the same prior is used but now observed trait CWM values belonging to specific genera are used as constraints (following earlier applications using simulated communities^11^). Third, observed regional abundances are used as prior with permutated trait constraints and finally both observed regional abundances and observed trait CWM are used as prior and constraints. *Maxent2*, an updated version of the *maxent* function currently in the FD library of R provided the computational platform. Proportions of uncertainty explained by each model are given by the Kullback–Leibler divergence R^2^_KL_, a generalization of the classic R^2^ goodness of fit. In contrast with standard linear regression models having squared goodness-of-fits measurements, the R^2^_KL_ is much more related to the concept of relative entropy, quantifying the information lost when one distribution is compared to another by means of quantifying the statistical distance between two distributions^40^. Pure trait, pure metacommunity, joint metacommunity-trait and unexplained effects are calculated as proportions of total biologically relevant information (Box S1 and Box S2). Data was rarefied to smallest sample size (swamp forests; 28) and calculations bootstrapped 25 times. Results indicated no significant change compared to using all data, hence the total dataset was used for all analyses.

### Strength and direction of selection

Predictions of genus relative abundances are computed as a function of traits reflected in the CWM values and a series of constants (λ_jk_: the Lagrange Multipliers). Each multiplier quantifies the association between a unit of change for a particular trait *j* and a proportional change in predicted relative abundance *p*_*ik*_ (the ith genus in the kth community) considering all other traits are constant, formally described as: $${\raise0.7ex\hbox{${ \partial p_{ik} }$} \!\mathord{\left/ {\vphantom {{ \partial p_{ik} } {\partial t_{ij} }}}\right.\kern-0pt} \!\lower0.7ex\hbox{${\partial t_{ij} }$}} = \lambda_{jk} p_{ik} \left( {1 - p_{ik} } \right)$$ (see appendix 1 from^41^). Positive values indicate larger trait values associated with higher abundances (positive selection), negative values indicate the opposite (negative selection) with changes proportional to lambda. Values approximating zero indicate no association between specific traits and relative abundances of species. Decomposing λ_jk_ and comparing by means of a One-Way Analysis of Variance for each trait separately between forest types allows studying both the strength and direction of selection in different habitats. Note that this is done for the same constraint between forest types, as lambda values for each constraint do not scale linearly between different constraints.

### Estimation of metacommunity size

Iteratively increasing the regional species pool considered as prior in concentric circles of a fixed radius of 50 km allows estimating the spatial effect of metacommunity size. Due to computational limits, the number of permutations for the MEF calculations (see above) was reduced to two, shuffling the combinations of genera and traits at least once. Comparison of results from the analyses using all plots indicated small effects of a smaller perturbations (average of 5% difference for metacommunity effect between 5 and 50 permutations). The relationship between pure metacommunity effect and radius of metacommunity size was fitted using a smoothing loess regression (function *loess* and *predict;* R-package *stats* with span set at 0.1). Fits subsequently were used to predict values of metacommunity effect based on geographical distance to visualize general patterns for each forest type. Exponential decay of pure metacommunity effect was described using a self-start asymptotic regression function (*SSasymp*) of the form *y(t)∼y*_*f*_ + (*y*_*0*_* − y*_*f*_)*e*^*−exp(log(α))t*^ (*nls* from *stats*^42^. A list of all packages used in R in addition to those preloaded can be found in the supplementary online material (SA2).

## Supplementary Information

Supplementary Information.

## Data Availability

R scripts are available on the github repository of E.T. Pos (EdwinTPos). The data that support the findings of this study are available from The Amazon Tree Diversity Network (ATDN) upon reasonable request.
