# Supplementary material for: Unraveling Amazon tree community assembly using Maximum Information Entropy: a quantitative analysis of tropical forest ecology
Source: Sci Rep. 2023 Feb 17;13:2859. doi: 10.1038/s41598-023-28132-y (PMC9938116; doi:10.1038/s41598-023-28132-y)
Supplement: Supplementary file 1 — Supplementary Information. [file 41598_2023_28132_MOESM1_ESM.pdf]

Supplementary information to the manuscript "Unraveling Amazon tree community assembly using Maximum Information Entropy: a quantitative analysis of tropical forest ecology".

Authors: Edwin Pos*,1,2 Luiz de Souza Coelho,3 Diogenes de Andrade Lima Filho,3 Rafael P. Salomão,4,5 Iêda Leão Amaral,3 Francisca Dionízia de Almeida Matos,3 Carolina V. Castilho,6 Oliver L. Phillips,7 Juan Ernesto Guevara,8,9 Marcelo de Jesus Veiga Carim,10 Dairon Cárdenas López,11 William E. Magnusson,12 Florian Wittmann,13,14 Mariana Victória Irume,3 Maria Pires Martins,3 Daniel Sabatier,15 José Renan da Silva Guimarães,10 Jean-François Molino,15 Olaf S. Bánki,2 Maria Teresa Fernandez Piedade,16 Nigel C.A. Pitman,17 Abel Monteagudo Mendoza,18 José Ferreira Ramos,3 Joseph E. Hawes,19 Everton José Almeida,20 Luciane Ferreira Barbosa,20 Larissa Cavalheiro,20 Márcia Cléia Vilela dos Santos,20 Bruno Garcia Luize,21 Evlyn Márcia Moraes de Leão Novo,22 Percy Núñez Vargas,23 Thiago Sanna Freire Silva,24 Eduardo Martins Venticinque,25 Angelo Gilberto Manzatto,26 Neidiane Farias Costa Reis,27 John Terborgh,28,29 Katia Regina Casula,27 Euridice N. Honorio Coronado,30,7 Juan Carlos Montero,31,3 Beatriz S. Marimon,32 Ben Hur Marimon-Junior,32 Ted R. Feldpausch,33,7 Alvaro Duque,34 Chris Baraloto,35 Nicolás Castaño Arboleda,11 Julien Engel,15,35 Pascal Petronelli,36 Charles Eugene Zartman,3 Timothy J. Killeen,37 Rodolfo Vasquez,18 Bonifacio Mostacedo,38 Rafael L. Assis,39 Jochen Schöngart,16 Hernán Castellanos,40 Marcelo Brilhante de Medeiros,41 Marcelo Fragomeni Simon,41 Ana Andrade,42 José Luís Camargo,42 Layon O. Demarchi,16 William F. Laurance,29 Susan G.W. Laurance,29 Emanuelle de Sousa Farias,43,44 Maria Aparecida Lopes,45 José Leonardo Lima Magalhães,46,47 Henrique Eduardo Mendonça Nascimento,3 Helder Lima de Queiroz,48 Gerardo A. Aymard C.,49 Roel Brienen,7 Juan David Cardenas Revilla,3 Flávia R.C. Costa,3 Adriano Quaresma,16 Ima Célia Guimarães Vieira,5 Bruno Barçante Ladvocat Cintra,50 Pablo R. Stevenson,51 Yuri Oliveira Feitosa,52 Joost F. Duivenvoorden,53 Hugo F. Mogollón,54 Leandro Valle Ferreira,5 James A. Comiskey,55,56 Freddie Draper,57,35 José Julio de Toledo,58 Gabriel Damasco,59 Nállarett Dávila,60 Roosevelt García-Villacorta,61,62 Aline Lopes,16,63 Alberto Vicentini,12 Janaína Costa Noronha,64 Flávia Rodrigues Barbosa,64 Rainiellen de Sá Carpanedo,64 Thaise Emilio,65,12 Carolina Levis,66,67 Domingos de Jesus Rodrigues,64 Juliana Schietti,3 Priscila Souza,3 Alfonso Alonso,56 Francisco Dallmeier,56 Vitor H.F. Gomes,68,69 Jon Lloyd,70 David Neill,71 Daniel Praia Portela de Aguiar,16 Alejandro Araujo-Murakami,72 Luzmila Arroyo,72 Fernanda Antunes Carvalho,12,73 Fernanda Coelho de Souza,12,7 Dário Dantas do Amaral,5 Kenneth J. Feeley,74,75 Rogerio Gribel,3 Marcelo Petratti Pansonato,3,76 Jos Barlow,77 Erika Berenguer,78,77 Joice Ferreira,47 Paul V.A. Fine,59 Marcelino Carneiro Guedes,79 Eliana M. Jimenez,80 Juan Carlos Licona,31 Maria Cristina Peñuela Mora,81 Carlos A. Peres,82 Boris Eduardo Villa Zegarra,83 Carlos Cerón,84 Terry W. Henkel,85 Paul Maas,2 Marcos Silveira,86 Juliana Stropp,87 Raquel Thomas-Caesar,88 Tim R. Baker,7 Doug Daly,89 Kyle G. Dexter,90,91 John Ethan Householder,13 Isau Huamantupa-Chuquimaco,23 Toby Pennington,33,91 Marcos Ríos Paredes,92 Alfredo Fuentes,93,94 José Luis Marcelo Pena,95 Miles R. Silman,96 J. Sebastián Tello,94 Jerome Chave,97 Fernando Cornejo Valverde,98 Anthony Di Fiore,99 Renato Richard Hilário,58 Juan Fernando Phillips,100 Gonzalo Rivas-Torres,101,102 Tinde R. van Andel,2,103 Patricio von Hildebrand,104 Edelcilio Marques Barbosa,3 Luiz Carlos de Matos Bonates,3 Hilda Paulette Dávila Doza,92 Émile Fonty,105,15 Ricardo Zárate Gómez,106 Therany Gonzales,107 George

Pepe Gallardo Gonzales,[92] Jean-Louis Guillaumet†,[108] Bruce Hoffman,[109] André Braga Junqueira,[110] Yadvinder Malhi,[111] Ires Paula de Andrade Miranda,[3] Linder Felipe Mozombite Pinto,[92] Adriana Prieto,[112] Agustín Rudas,[112] Ademir R. Ruschel,[47] Natalino Silva,[113] César I.A. Vela,[114] Vincent Antoine Vos,[115] Egleé L. Zent,[116] Stanford Zent,[116] Bianca Weiss Albuquerque,[16] Angela Cano,[51,117] Diego F. Correa,[51,118] Janaina Barbosa Pedrosa Costa,[79] Bernardo Monteiro Flores,[119] Milena Holmgren,[120] Marcelo Trindade Nascimento,[121] Alexandre A. Oliveira,[76] Hirma Ramirez-Angulo,[122] Maira Rocha,[16] Veridiana Vizoni Scudeller,[123] Rodrigo Sierra,[124] Milton Tirado,[124] Maria Natalia Umaña,[125] Geertje van der Heijden,[126] Emilio Vilanova Torre,[122,127] Corine Vriesendorp,[17] Ophelia Wang,[128] Kenneth R. Young,[129] Manuel Augusto Ahuite Reategui,[130] Cláudia Baider,[131,76] Henrik Balslev,[132] Sasha Cárdenas,[51] Luisa Fernanda Casas,[51] William Farfan-Rios,[23,133,94] Cid Ferreira,[3] Reynaldo Linares-Palomino,[56] Casimiro Mendoza,[134,135] Italo Mesones,[59] Armando Torres-Lezama,[122] Ligia Estela Urrego Giraldo,[34] Daniel Villarroel,[72] Roderick Zagt,[136] Miguel N. Alexiades,[137] Karina Garcia-Cabrera,[96] Lionel Hernandez,[40] William Milliken,[65] Walter Palacios Cuenca,[138] Susamar Pansini,[27] Daniela Pauletto,[139] Freddy Ramirez Arevalo,[140] Adeilza Felipe Sampaio,[27] Elvis H. Valderrama Sandoval,[141,140] Luis Valenzuela Gamarra,[18] Gerhard Boenisch,[142] Jens Kattge,[143] Nathan Kraft,[144] Aurora Levesley,[7] Karina Melgaço,[7] Georgia Pickavance,[7] Lourens Poorter,[67] Hans ter Steege,[2]

* Corresponding author: e.t.pos@uu.nl
† Deceased 01-2018

Affiliations
1 Quantitative Biodiversity Dynamics, Ecology & Biodiversity & Utrecht University Botanic Gardens, Utrecht University, Padualaan 8, Utrecht, 3584 CH, The Netherlands
2Naturalis Biodiversity Center, PO Box 9517, Leiden, 2300 RA, The Netherlands
3Coordenação de Biodiversidade, Instituto Nacional de Pesquisas da Amazônia - INPA, Av. André Araújo, 2936, Petrópolis, Manaus, AM, 69067-375, Brazil
4Programa Professor Visitante Nacional Sênior na Amazônia - CAPES, Universidade Federal Rural da Amazônia, Av. Perimetral, s/n, Belém, PA, Brazil
5Coordenação de Botânica, Museu Paraense Emílio Goeldi, Av. Magalhães Barata 376, C.P. 399, Belém, PA, 66040-170, Brazil
6EMBRAPA – Centro de Pesquisa Agroflorestal de Roraima, BR 174, km 8 – Distrito Industrial, Boa Vista, RR, 69301-970, Brazil
7School of Geography, University of Leeds, Woodhouse Lane, Leeds, LS2 9JT, UK
8Grupo de Investigación en Biodiversidad, Medio Ambiente y Salud-BIOMAS, Universidad de las Américas, Campus Queri, Quito, Ecuador
9Keller Science Action Center, The Field Museum, 1400 S. Lake Shore Drive, Chicago, IL, 60605-2496, USA
10Departamento de Botânica, Instituto de Pesquisas Científicas e Tecnológicas do Amapá - IEPA, Rodovia JK, Km 10, Campus do IEPA da Fazendinha, Amapá, 68901-025, Brazil
11Herbario Amazónico Colombiano, Instituto SINCHI, Calle 20 No 5-44, Bogotá, DC, Colombia
12Coordenação de Pesquisas em Ecologia, Instituto Nacional de Pesquisas da Amazônia - INPA, Av. André Araújo, 2936, Petrópolis, Manaus, AM, 69067-375, Brazil
13Dep. of Wetland Ecology, Institute of Geography and Geoecology, Karlsruhe Institute of Technology - KIT, Josefstr.1, Rastatt, D-76437, Germany
14Biogeochemistry, Max Planck Institute for Chemistry, Hahn-Meitner Weg 1, Mainz, 55128, Germany
15AMAP, IRD, Cirad, CNRS, INRA, Université de Montpellier, Montpellier, F-34398, France
16Coordenação de Dinâmica Ambiental, Instituto Nacional de Pesquisas da Amazônia - INPA, Av. André Araújo, 2936, Petrópolis, Manaus, AM, 69067-375, Brazil
17Science and Education, The Field Museum, 1400 S. Lake Shore Drive, Chicago, IL, 60605-2496, USA
18Jardín Botánico de Missouri, Oxapampa, Pasco, Peru
19Applied Ecology Research Group, School of Life Sciences, Anglia Ruskin University, East Road, Cambridge, CB1 1PT, UK
20ICNHS, Universidade Federal de Mato Grosso, Av. Alexandre Ferronato, 1200, Sinop, MT, 78557-267, Brazil
21Departamento de Ecologia, Universidade Estadual Paulista - UNESP – Instituto de Biociências – IB, Av. 24 A, 1515, Bela Vista, Rio Claro, SP, 13506-900, Brazil
22Divisao de Sensoriamento Remoto – DSR, Instituto Nacional de Pesquisas Espaciais – INPE, Av. dos Astronautas, 1758, Jardim da Granja, São José dos Campos, SP, 12227-010, Brazil
23Herbario Vargas, Universidad Nacional de San Antonio Abad del Cusco, Avenida de la Cultura, Nro 733, Cusco, Cuzco, Peru
24Biological and Environmental Sciences, University of Stirling, Stirling, FK9 4LA, UK
25Centro de Biociências, Departamento de Ecologia, Universidade Federal do Rio Grande do Norte, Av. Senador Salgado Filho, 3000 , Natal, RN, 59072-970, Brazil

[26]Departamento de Biologia, Universidade Federal de Rondônia, Rodovia BR 364 s/n Km 9,5 - Sentido Acre, Unir, Porto Velho, RO, 76.824-027, Brazil
[27]Programa de Pós- Graduação em Biodiversidade e Biotecnologia PPG- Bionorte, Universidade Federal de Rondônia, Campus Porto Velho Km 9,5 bairro Rural, Porto Velho, RO, 76.824-027, Brazil
[28]Department of Biology and Florida Museum of Natural History, University of Florida, Gainesville, FL, 32611, USA
[29]Centre for Tropical Environmental and Sustainability Science and College of Science and Engineering, James Cook University, Cairns, Queensland, 4870, Australia
[30]Instituto de Investigaciones de la Amazonía Peruana (IIAP), Av. A. Quiñones km 2,5, Iquitos, Loreto, 784, Peru
[31]Instituto Boliviano de Investigacion Forestal, Av. 6 de agosto #28, Km. 14, Doble via La Guardia, Casilla 6204, Santa Cruz, Santa Cruz, Bolivia
[32]Programa de Pós-Graduação em Ecologia e Conservação, Universidade do Estado de Mato Grosso, Nova Xavantina, MT, Brazil
[33]Geography, College of Life and Environmental Sciences, University of Exeter, Rennes Drive, Exeter, EX4 4RJ, UK
[34]Departamento de Ciencias Forestales, Universidad Nacional de Colombia, Calle 64 x Cra 65, Medellín, Antioquia, 1027, Colombia
[35]International Center for Tropical Botany (ICTB) Department of Biological Sciences, Florida International University, 11200 SW 8th Street, OE 243, Miami, FL, 33199, USA
[36]Cirad UMR Ecofog, AgrosParisTech,CNRS,INRA,Univ Guyane, Campus agronomique, Kourou Cedex, 97379, France
[37]Agteca-Amazonica, Santa Cruz, Bolivia
[38]Facultad de Ciencias Agrícolas, Universidad Autónoma Gabriel René Moreno, Santa Cruz, Santa Cruz, Bolivia
[39]Natural History Museum, University of Oslo, Postboks 1172, Oslo, 0318, Norway
[40]Centro de Investigaciones Ecológicas de Guayana, Universidad Nacional Experimental de Guayana, Calle Chile, urbaniz Chilemex, Puerto Ordaz, Bolivar, Venezuela
[41]Prédio da Botânica e Ecologia, Embrapa Recursos Genéticos e Biotecnologia, Parque Estação Biológica, Av. W5 Norte, Brasilia, DF, 70770-917, Brazil
[42]Projeto Dinâmica Biológica de Fragmentos Florestais, Instituto Nacional de Pesquisas da Amazônia - INPA, Av. André Araújo, 2936, Petrópolis, Manaus, AM, 69067-375, Brazil
[43]Laboratório de Ecologia de Doenças Transmissíveis da Amazônia (EDTA), Instituto Leônidas e Maria Deane, Fiocruz, Rua Terezina, 476, Adrianópolis, Manaus, AM, 69060-001, Brazil
[44]Programa de Pós-graduação em Biodiversidade e Saúde, Instituto Oswaldo Cruz - IOC/FIOCRUZ, Pav. Arthur Neiva – Térreo, Av. Brasil, 4365 – Manguinhos, Rio de Janeiro, RJ, 21040-360, Brazil
[45]Instituto de Ciências Biológicas, Universidade Federal do Pará, Av. Augusto Corrêa 01, Belém, PA, 66075-110, Brazil
[46]Programa de Pós-Graduação em Ecologia, Universidade Federal do Pará, Av. Augusto Corrêa 01, Belém, PA, 66075-110, Brazil
[47]Embrapa Amazônia Oriental, Trav. Dr. Enéas Pinheiro s/nº, Belém, PA, 66095-100, Brazil
[48]Diretoria Técnico-Científica, Instituto de Desenvolvimento Sustentável Mamirauá, Estrada do Bexiga, 2584, Tefé, AM, 69470-000, Brazil

49Programa de Ciencias del Agro y el Mar, Herbario Universitario (PORT), UNELLEZ-Guanare, Guanare, Portuguesa, 3350, Venezuela
50Instituto de Biociências - Dept. Botanica, Universidade de Sao Paulo - USP, Rua do Matão 277, Cidade Universitária, São Paulo, SP, 05508-090, Brazil
51Laboratorio de Ecología de Bosques Tropicales y Primatología, Universidad de los Andes, Carrera 1 # 18a- 10, Bogotá, DC, 111711, Colombia
52Programa de Pós-Graduação em Biologia (Botânica), Instituto Nacional de Pesquisas da Amazônia - INPA, Av. André Araújo, 2936, Petrópolis, Manaus, AM, 69067-375, Brazil
53Institute of Biodiversity and Ecosystem Dynamics, University of Amsterdam, Sciencepark 904, Amsterdam, 1098 XH, The Netherlands
54Endangered Species Coalition, 8530 Geren Rd., Silver Spring, MD, 20901, USA
55Inventory and Monitoring Program, National Park Service, 120 Chatham Lane, Fredericksburg, VA, 22405, USA
56Center for Conservation and Sustainability, Smithsonian Conservation Biology Institute, 1100 Jefferson Dr. SW, Suite 3123, Washington, DC, 20560-0705, USA
57Department of Global Ecology, Carnegie Institution for Science, 260 Panama St., Stanford, CA, 94305, USA
58Universidade Federal do Amapá, Ciências Ambientais, Rod. Juscelino Kubitschek km2, Macapá, AP, 68902-280, Brazil
59Department of Integrative Biology, University of California, Berkeley, CA, 94720-3140, USA
60Biologia Vegetal, Universidade Estadual de Campinas, Caixa Postal 6109, Campinas, SP, 13.083-970, Brazil
61Department of Ecology and Evolutionary Biology, Cornell University, Corson Hall, 215 Tower Road, Ithaca, NY, 14850, USA
62Peruvian Center for Biodiversity and Conservation (PCBC), Iquitos, Peru
63Department of Ecology, University of Brasilia, Brasilia, DF, 70904-970, Brazil
64ICNHS, Federal University of Mato Grosso, Av. Alexandre Ferronato 1200, Setor Industrial, Sinop, MT, 78.557-267, Brazil
65Natural Capital and Plant Health, Royal Botanic Gardens, Kew, Richmond, Surrey, TW9 3AB, UK
66Programa de Pós-Graduação em Ecologia, Instituto Nacional de Pesquisas da Amazônia - INPA, Av. André Araújo, 2936, Petrópolis, Manaus, AM, 69067-375, Brazil
67Forest Ecology and Forest Management Group, Wageningen University & Research, Droevendaalsesteeg 3, Wageningen, P.O. Box 47, 6700 AA, The Netherlands
68Escola de Negócios Tecnologia e Inovação, Centro Universitário do Pará, Belém, PA, Brazil
69Universidade Federal do Pará, Rua Augusto Corrêa 01, Belém, PA, 66075-110, Brazil
70Faculty of Natural Sciences, Department of Life Sciences, Imperial College London, Silwood Park, South Kensington Campus, London, SW7 2AZ, UK
71Ecosistemas, Biodiversidad y Conservación de Especies, Universidad Estatal Amazónica, Km. 2 1/2 vía a Tena (Paso Lateral), Puyo, Pastaza, Ecuador
72Museo de Historia Natural Noel Kempff Mercado, Universidad Autónoma Gabriel Rene Moreno, Avenida Irala 565 Casilla Post al 2489, Santa Cruz, Santa Cruz, Bolivia
73Universidade Federal de Minas Gerais, Instituto de Ciências Biológicas, Departamento de Genética, Ecologia e Evolução, Av. Antônio Carlos, 6627 Pampulha, Belo Horizonte, MG, 31270-901, Brazil
74Department of Biology, University of Miami, Coral Gables, FL, 33146, USA

75Fairchild Tropical Botanic Garden, Coral Gables, FL, 33156, USA
76Instituto de Biociências - Dept. Ecologia, Universidade de Sao Paulo - USP, Rua do Matão, Trav. 14, no. 321, Cidade Universitária, São Paulo, SP, 05508-090, Brazil
77Lancaster Environment Centre, Lancaster University, Lancaster, Lancashire, LA1 4YQ, UK
78Environmental Change Institute, University of Oxford, Oxford, Oxfordshire, OX1 3QY, UK
79Empresa Brasileira de Pesquisa Agropecuária, Embrapa Amapá, Rod. Juscelino Kubitschek km 5, Macapá, Amapá, 68903-419, Brazil
80Grupo de Ecología y Conservación de Fauna y Flora Silvestre, Instituto Amazónico de Investigaciones Imani, Universidad Nacional de Colombia sede Amazonia, Leticia, Amazonas, Colombia
81Universidad Regional Amazónica IKIAM, Km 7 via Muyuna, Tena, Napo, Ecuador
82School of Environmental Sciences, University of East Anglia, Norwich, NR4 7TJ, UK
83Direccíon de Evaluación Forestal y de Fauna Silvestre, Av. Javier Praod Oeste 693, Magdalena del Mar, Peru
84Escuela de Biología Herbario Alfredo Paredes, Universidad Central, Ap. Postal 17.01.2177, Quito, Pichincha, Ecuador
85Department of Biological Sciences, Humboldt State University, 1 Harpst Street, Arcata, CA, 95521, USA
86Museu Universitário / Centro de Ciências Biológicas e da Natureza / Laboratório de Botânica e Ecologia Vegetal, Universidade Federal do Acre, Rio Branco, AC, 69915-559, Brazil
87Institute of Biological and Health Sciences, Federal University of Alagoas, Av. Lourival Melo Mota, s/n, Tabuleiro do Martins, Maceio, AL, 57072-970, Brazil
88Iwokrama International Centre for Rain Forest Conservation and Development, Georgetown, Guyana
89New York Botanical Garden, 2900 Southern Blvd, Bronx, New York, NY, 10458-5126, USA
90School of Geosciences, University of Edinburgh, 201 Crew Building, King's Buildings, Edinburgh, EH9 3JN, UK
91Tropical Diversity Section, Royal Botanic Garden Edinburgh, 20a Inverleith Row, Edinburgh, Scotland, EH3 5LR, UK
92Servicios de Biodiversidad EIRL, Jr. Independencia 405, Iquitos, Loreto, 784, Peru
93Herbario Nacional de Bolivia, Universitario UMSA, Casilla 10077 Correo Central, La Paz, La Paz, Bolivia
94Center for Conservation and Sustainable Development, Missouri Botanical Garden, P.O. Box 299, St. Louis, MO, 63166-0299, USA
95Universidad Nacional de Jaén, Carretera Jaén San Ignacio Km 23, Jaén, Cajamarca, 06801, Peru
96Biology Department and Center for Energy, Environment and Sustainability, Wake Forest University, 1834 Wake Forest Rd, Winston Salem, NC, 27106, USA
97Laboratoire Evolution et Diversité Biologique, CNRS and Université Paul Sabatier, UMR 5174 EDB, Toulouse, 31000, France
98Andes to Amazon Biodiversity Program, Madre de Dios, Madre de Dios, Peru
99Department of Anthropology, University of Texas at Austin, SAC 5.150, 2201 Speedway Stop C3200, Austin, TX, 78712, USA
100Fundación Puerto Rastrojo, Cra 10 No. 24-76 Oficina 1201, Bogotá, DC, Colombia
101Colegio de Ciencias Biológicas y Ambientales-COCIBA & Galapagos Institute for the Arts and Sciences-GAIAS, Universidad San Francisco de Quito-USFQ, Quito, Pichincha, Ecuador

102Department of Wildlife Ecology and Conservation, University of Florida, 110 Newins-Ziegler Hall, Gainesville, FL, 32611, USA
103Biosystematics group, Wageningen University, Droevendaalsesteeg 1, Wageningen, 6708 PB, The Netherlands
104Fundación Estación de Biología, Cra 10 No. 24-76 Oficina 1201, Bogotá, DC, Colombia
105Direction régionale de la Guyane, ONF, Cayenne, F-97300, French Guiana
106PROTERRA, Instituto de Investigaciones de la Amazonía Peruana (IIAP), Av. A. Quiñones km 2,5, Iquitos, Loreto, 784, Peru
107ACEER Foundation, Jirón Cusco N° 370, Puerto Maldonado, Madre de Dios, Peru
108Departement EV, Muséum national d'histoire naturelle de Paris, 16 rue Buffon, Paris, 75005, France
109Amazon Conservation Team, Doekhieweg Oost #24, Paramaribo, Suriname
110Institut de Ciència i Tecnologia Ambientals, Universitat Autònoma de Barcelona, 08193 Bellaterra, Barcelona, Spain
111Environmental Change Institute, Oxford University Centre for the Environment, Dyson Perrins Building, South Parks Road, Oxford, England, OX1 3QY, UK
112Instituto de Ciencias Naturales, Universidad Nacional de Colombia, Apartado 7945, Bogotá, DC, Colombia
113Instituto de Ciência Agrárias, Universidade Federal Rural da Amazônia, Av. Presidente Tancredo Neves 2501, Belém, PA, 66.077-830, Brazil
114Escuela Profesional de Ingeniería Forestal, Universidad Nacional de San Antonio Abad del Cusco, Jirón San Martín 451, Puerto Maldonado, Madre de Dios, Peru
115Universidad Autónoma del Beni José Ballivián, Campus Universitario Final, Av. Ejercito, Riberalta, Beni, Bolivia
116Laboratory of Human Ecology, Instituto Venezolano de Investigaciones Científicas - IVIC, Ado 20632, Caracas, DC, 1020A, Venezuela
117Cambridge University Botanic Garden, 1 Brookside., Cambridge, CB2 1JE, UK
118School of Agriculture and Food Sciences - ARC Centre of Excellence for Environmental Decisions CEED, The University of Queensland, St. Lucia, QLD 4072, Australia
119University of Campinas, Plant Biology Department, Rua Monteiro Lobato, 255, Cidade Universitária Zeferino Vaz, Barão Geraldo, Campinas, São Paulo, CEP 13083-862, Brazil
120Resource Ecology Group, Wageningen University & Research, Droevendaalsesteeg 3a, Lumen, building number 100, Wageningen, Gelderland, 6708 PB, The Netherlands
121Laboratório de Ciências Ambientais, Universidade Estadual do Norte Fluminense, Av. Alberto Lamego 2000, Campos dos Goyatacazes, RJ, 28013-620, Brazil
122Instituto de Investigaciones para el Desarrollo Forestal (INDEFOR), Universidad de los Andes, Conjunto Forestal, 5101, Mérida, Mérida, Venezuela
123Departamento de Biologia, Universidade Federal do Amazonas - UFAM – Instituto de Ciências Biológicas – ICB1, Av General Rodrigo Octavio 6200, Manaus, AM, 69080-900, Brazil
124GeoIS, El Día 369 y El Telégrafo, 3° Piso, Quito, Pichincha, Ecuador
125Department of Ecology and Evolutionary Biology, University of Michigan, Ann Arbor, MI, 48109, USA
126University of Nottingham, University Park, Nottingham, NG7 2RD, UK
127School of Environmental and Forest Sciences, University of Washington, Seattle, WA, 98195-2100, USA

128Environmental Science and Policy, Northern Arizona University, Flagstaff, AZ, 86011, USA
129Geography and the Environment, University of Texas at Austin, 305 E. 23rd Street, CLA building, Austin, TX, 78712, USA
130Medio Ambiente, PLUSPRETOL, Iquitos, Loreto, Peru
131The Mauritius Herbarium, Agricultural Services, Ministry of Agro-Industry and Food Security, Reduit, 80835, Mauritius
132Department of Bioscience, Aarhus University, Building 1540 Ny Munkegade, Aarhus C, Aarhus, DK-8000, Denmark
133Living Earth Collaborative, Washington University in Saint Louis, St. Louis, MO, 63130, USA
134Escuela de Ciencias Forestales (ESFOR), Universidad Mayor de San Simon (UMSS), Sacta, Cochabamba, Bolivia
135FOMABO, Manejo Forestal en las Tierras Tropicales de Bolivia, Sacta, Cochabamba, Bolivia
136Tropenbos International, Lawickse Allee 11 PO Box 232, Wageningen, 6700 AE, The Netherlands
137School of Anthropology and Conservation, University of Kent, Marlowe Building, Canterbury, Kent, CT2 7NR, UK
138Herbario Nacional del Ecuador, Universidad Técnica del Norte, Quito, Pichincha, Ecuador
139Instituto de Biodiversidade e Floresta, Universidade Federal do Oeste do Pará, Rua Vera Paz, Campus Tapajós, Santarém, PA, 68015-110, Brazil
140Facultad de Biologia, Universidad Nacional de la Amazonia Peruana, Pevas 5ta cdra, Iquitos, Loreto, Peru
141Department of Biology, University of Missouri, St. Louis, MO, 63121, USA
142Department of Biogeochemical Integration, Max-Planck-Institute for Biogeochemistry, P.O. Box 10 01 64, Jena, 07701, Germany
143Functional Biogeography, Max-Planck-Institute for Biogeochemistry, P.O. Box 10 01 64, Jena, 07701, Germany
144Department of Ecology and Evolutionary Biology, UCLA, 621 Charles E. Young Drive South, Box 951606, Los Angeles, CA, 90095, USA

Contents of Supplementary Material

1. Box S1-S3
2. Figures S1-8
3. Tables S1-S2
4. Ecological interpretation of the MEF results (S-A)
5. List of packages used
6. References for SI

BOX S1

Entities:
The basic unit of the MEF model which can exist in different states. Here, this constitutes a collection of genera existing at a site, hence each entity can be considered a single genus.

States:
Classification of different ways any entity can exist. Within the system, states of each entity describe their specific abundance at that site. Microstates constitute the spatial and temporal composition for the states of the entities in the system. Macrostates depict the entities of a system independent of the spatial or temporal composition, e.g., the overall relative abundance distribution but not including processes leading to this distribution (such as dispersal).

Traits or properties:
The measurable attributes for each entity, of which the values can be different for each entity. For example, genera differ in average wood density, seed mass, height etcetera. Here defined by the functional traits as described in the main text.

Maximally uninformative prior:
All the information regarding the states before specific constraints are introduced. Described as maximally uninformative as all empirical information should be introduced in the form of constraints quantify the maximal gain of information regarding the different constraints (e.g. traits or prior distribution in this case).

Prior distribution:
The expected states for the entities, here either constituted by the observed relative abundance of each entity in the summed sample (i.e. summed abundances describing the metacommunity) or by a maximally uninformed (uniform) distribution (see above). The former would be a neutral prior (expected local abundance is equal to the abundance in the larger metacommunity).

Community-weighted mean or variance:
The mean or variance of genus-level trait value over all constituent present species (for each entity) weighted by the relative abundance of each entity at a specific site.

Box S1. Different ingredients necessary for analyses using MEF. Definitions of the most important terms used in the MEF analyses and throughout the main text to provide the necessary framework of understanding, adapted from [1].

BOX S2

The Maximum Entropy Formalism as applied here works based on a conceptual model called CATS (*Community Assembly by Trait Selection* [1–3]) and makes use of three inputs:

i) A **trait matrix** containing the measured functional traits of each of the S total genera in the total regional pool, these can be of either discrete or continuous form.

ii) A **vector of *n* community weighted trait values**, estimating the average trait value over all individuals in the local community for each of the traits

iii) A **prior probability distribution** specifying the regional abundance distribution, quantifying potential contributions of the regional pool of recruits to the structure of local communities. Using these three sources of information, the model predicts relative abundances (p_i) in the form of Bayesian probabilities for each genus in each local community without assuming any a priori relations or processes. This is achieved by finding the vector of relative abundances maximizing entropy:

$$1)\ RE = -\sum_{i=1}^{S} p_i \ln\left(\frac{p_i}{q_i}\right)$$

with q_i the regional species pool abundance of species *i* and *RE* (Relative Entropy) subject to the known constraints for *j* traits and *i* species.:

$$2)\ \bar{t_j} = \sum_{i=1}^{S} o_i t_{ij} \text{ and } 3)\ \sum_{i=1}^{S} p_i = 1$$

The solution is a generalized exponential distribution where the λ values measure the importance of each trait when all other traits are constant:

$$4)\ p_i = \frac{q_i e^{\sum_{j=1}^{n}\lambda_j t_{ij}}}{\sum_{i=1}^{s} q_i e^{\sum_{j=1}^{T}\lambda_j t_{ij}}}$$

Note that when all λ values are zero, i.e. there is no trait based selection, $p_i = q_i$

The final step is to measure the proportion of total deviance accounted for between observed and predicted relative abundances for each of the four-step solution. These are the R^2_{KL} values, a generalization of the classic R^2 index of maximum likelihood estimation using the Kullback-Leibler index [4,5]:

i) **$\bar{R}^2_{KL}(u)$:** fit of model bias, the model null hypotheses given a uniform prior (i.e. equal distribution in the regional pool of recruits).

ii) **$R^2_{KL}(u, t)$:** fit using again a uniform prior but including traits as constraints.

iii) **$\bar{R}^2_{KL}(m)$:** fit using the metacommunity prior but excluding traits as constraints

iv) **$R^2_{KL}(m, t)$:** fit using the metacommunity prior and including traits as constraints

The general form of the **R^2_{KL}** divergence is calculated by:

$$5)\ R^2{}_{KL} = 1 - \frac{\sum_{k=1}^{c}\sum_{i=1}^{S} O_{ik}\ln\left(\frac{O_{ik}}{P_{ik}}\right)}{\sum_{j=k}^{c}\sum_{i=1}^{S} O_{ik}\ln\left(\frac{O_{ij}}{Q_{i,0}}\right)}$$

With the following parameters:

O_{ik} as the observed relative abundances of the ith genus in the kth community,

P_{ik} the accompanying predicted values for the specific model of the four solution step as described in the main text and,

$Q_{i,0}$ the predicted relative abundances given only the maximum uninformative prior.

Further details on the calculation of all separate **R^2_{KL}** values and accompanying pure trait, pure metacommunity, joint information and biologically unexplained information can be found Box S3.

Box S2. Mathematical description of the Maximum Entropy Formalism for the four-step solution. Left panel shows necessary ingredients and formulation of the Maximum Entropy Formalism. Right side panel shows decomposition of the proportion of total deviance accounted for between observed and predicted relative abundances for each of the four-step solution, adapted from [5].

BOX S3

The purpose of using MEF is to decompose the deviance between observed and predicted relative abundances using the four-step solution as described in the main text. The values generated are described below. The R^2_{KL} value is a generalization of the classic R^2 index of maximum likelihood estimation using the Kullback-Leibler index for a non-linear regression including a multinomial error structure [2,4,5]. *In essence, it is a way of measuring the proportion of total deviance accounted for by that specific model from one of the four steps:*

$\bar{R}^2_{KL}(u)$: fit of model bias, the model null hypotheses given a uniform prior and permuted traits
$R^2_{KL}(u, t)$: fit using a uniform prior but including observed traits as constraints
$\bar{R}^2_{KL}(m)$: fit using the metacommunity prior but excluding observed traits as constraints
$R^2_{KL}(m, t)$: fit using the metacommunity prior and including observed traits as constraints

1) The increase in the explained deviance due to traits can be calculated either by

$\Lambda R^2_{KL}(t|\varphi) = R^2_{KL}(u, t) - \bar{R}^2_{KL}(u)$
Increase in explained deviance due to traits beyond that due solely to model bias

or **$\Lambda R^2_{KL}(t|m) = R^2_{KL}(m, t) - \bar{R}^2_{KL}(m)$**
Increase in explained deviance due to traits beyond contributions made by the meta-community

2) The increase in explained deviance due dispersal mass effects via the metacommunity can be calculated by either:

$\Lambda R^2_{KL}(m|\ \varphi) = \bar{R}^2_{KL}(m) - \bar{R}^2_{KL}(u)$
Increase in explained deviance (if any) due to the metacommunity beyond that due to model bias

or **$\Lambda R^2_{KL}(m|t) = R^2_{KL}(m, t) - R^2_{KL}(u, t)$**
Increase in explained deviance due to the meta-community given traits, relative to the explained deviance due only to the traits: i.e. information unique to neutral prior

3) And finally the joint information and the biologically unexplained information:

$\Lambda R^2_{KL}(m+t) = \Lambda R^2_{KL}(m|\ \varphi) - \Lambda R^2_{KL}(m|t) = \Lambda R^2_{KL}(t|\varphi) - \Lambda R^2_{KL}(t|m)$
Joint information gain, or increase in explained deviance due to both the metacommunity prior and the constraints based on the traits

1- **$\Lambda R^2_{KL}(m,t)$**
Biologically unexplained variation

From these values the pure trait, pure metacommunity, joint effect and biologically unexplained variation can be calculated by the following calculations:

Pure trait effects: **$\Lambda R^2_{KL}(t|m)\ /\ (1-\bar{R}^2_{KL}(u))$**
Pure metacommunity effects: **$\Lambda R^2_{KL}(m|t)\ /\ (1-\bar{R}^2_{KL}(u))$**
Joint metacommunity and trait effects: **$\Lambda R^2_{KL}(m+t)\ /\ (1-\bar{R}^2_{KL}(u))$**
Unexplained effects: 1-**$\Lambda R^2_{KL}(m,t)\ /\ (1-\bar{R}^2_{KL}(u))$**

Box S3. Detailed decomposition of the four-step solution from the MEF. Mathematical description of the decomposition based on the constraints and prior distributions (both uniform and neutral) for each of the steps from the four-step solution to measure the proportion of total deviance accounted for by each specific model from one of the four steps, adapted from [5].

FIGURE S1

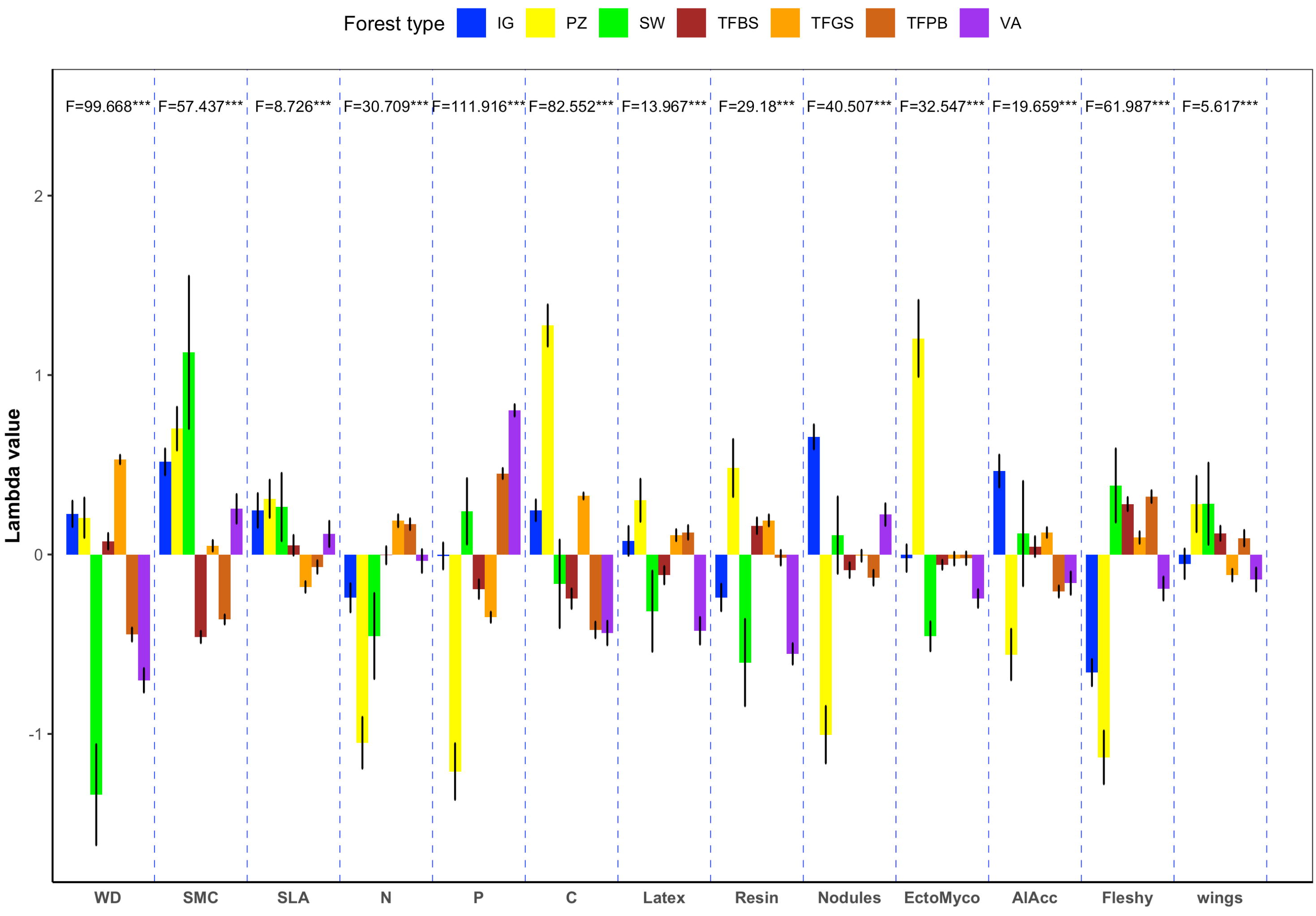

Fig. S1. Mean lambda values with standard error bars for each functional trait and compared between forest types. Forest type abbreviations are *igapó* (IG), podzol (PZ), swamp (SW), Brazilian shield *terra firme* (TFBS), Guiana Shield *terra* firme (TFGS), Pebas *terra* firme (TFPB) and *várzea* (VA). Positive values indicate positive selection, reflective of a strong association between higher trait values and higher abundances, negative values reflect the opposite with high trait values associated with lower abundances. Differences between forest types were tested with a one-way analysis of variance with significance levels corresponding to: ns non-significant, * $p < .05$, ** $p < .01$ and *** $p < .001$. Abbreviations indicate functional traits: wood density (WD), seed mass class (SMC), specific leaf area (SLA), nitrogen (N), phosphorus (P) and carbon (C) leaf content. Further traits include the presence/absence of Latex, Resin, Nodules, Ectomycorrhiza (EctoMyco), the ability to accumulate aluminium (AlAcc), and the presence/absence of fleshy fruits (Fleshy) and winged seeds (Wings).

FIGURE S2

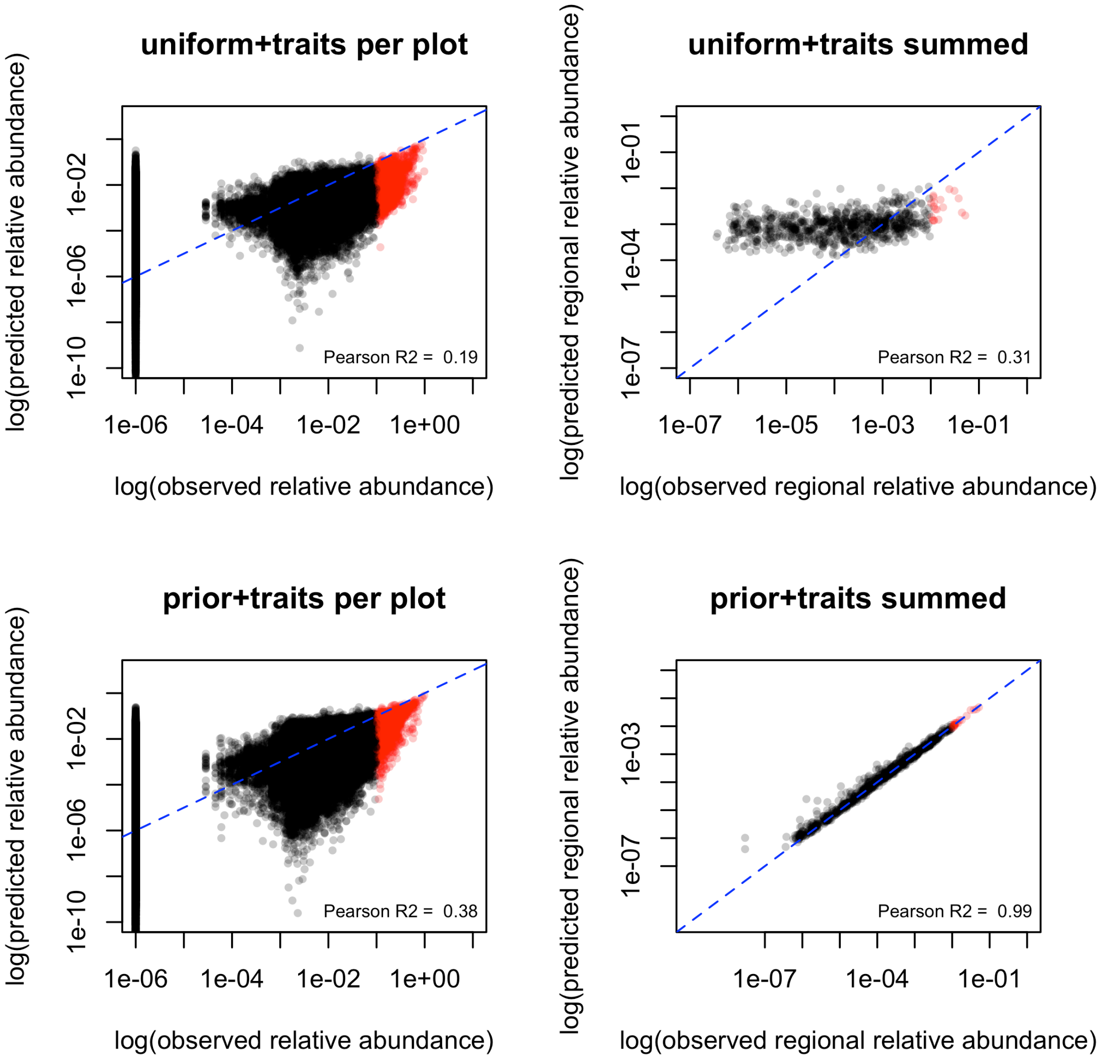

Fig. S2. Observed relative abundances for each genus in all plots plotted against predicted relative abundance per plot (left) and summed (right) using only the traits as constraints in combination with a uniform prior (top) or the hybrid model using both traits and the metacommunity relative abundance as prior (bottom) on a log-log scale. Top figures show predictions using only a uniform prior, left separate for all plots and right for all genera summed over all plots. Bottom figures show predictions using the regional prior, again separate for all plots and genera (left) and summed over all plots for each genus (right). Red points indicate taxa with observed relative abundances over 1e-1. Lines show the x=y prediction and R^2 values correspond to the Pearson's correlation coefficient.

FIGURE S3

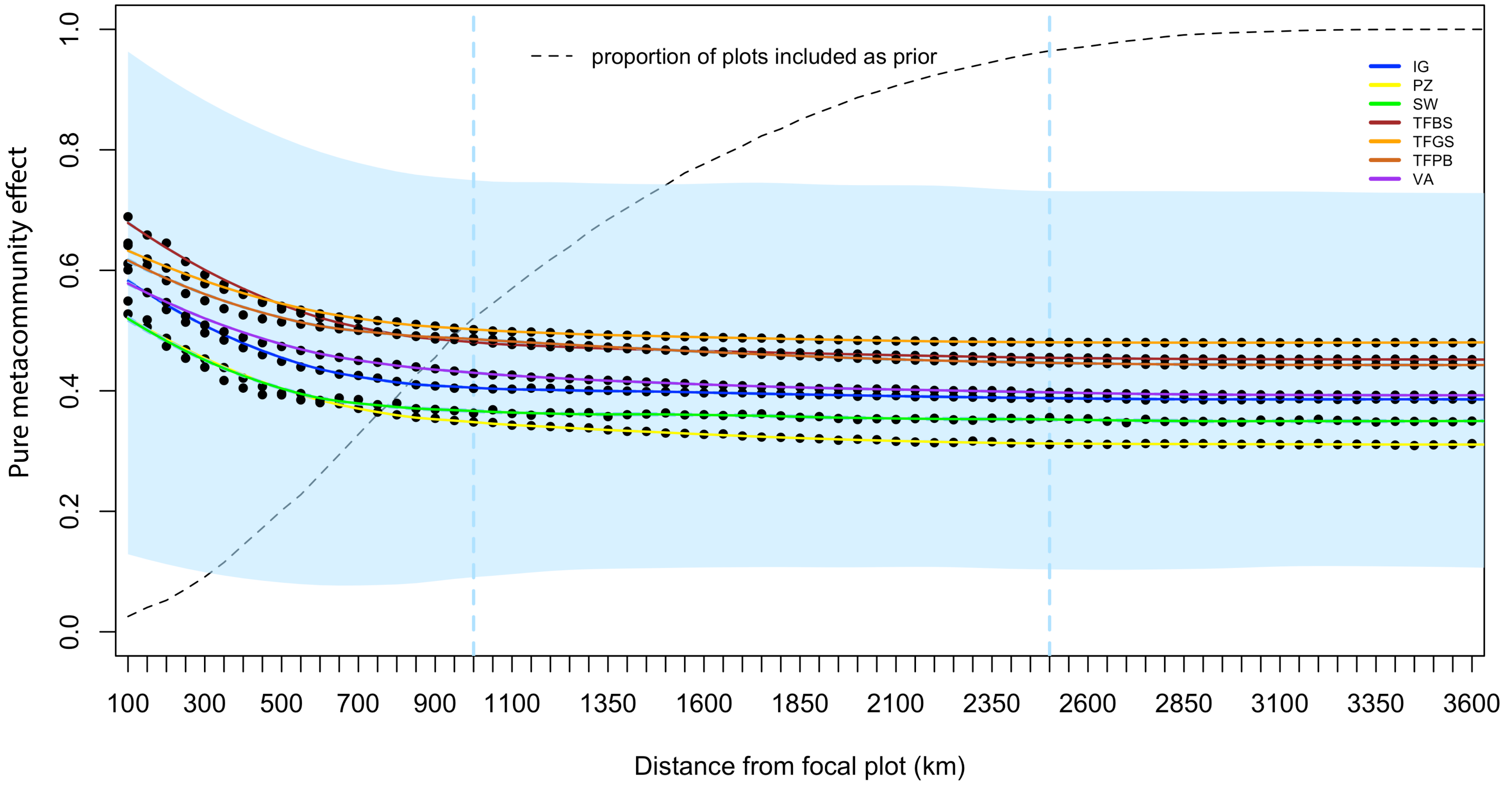

Fig. S3. Distance decay of pure metacommunity effect. X-axis represents radius of metacommunity prior; i.e. first 100 km consists of just a few plots and at 3800 km all plots are taken into account with dashed line indicating the mean number of plots for that distance included as metacommunity prior. Y-axis represents the pure metacommunity effect, i.e. the increase in explained deviance due to the metacommunity (given traits), but relative to the explained deviance due only to the traits. It is the information unique to neutral prior taken relative to the model bias. Solid lines indicate predictions from loess regression based on all points with different colors indicating the forest types with abbreviations as in main text. Blue vertical lines indicate 1000 and 2500 km boundary points. Blue shading reflects minimum and maximum loess regression predicted values.

FIGURE S4

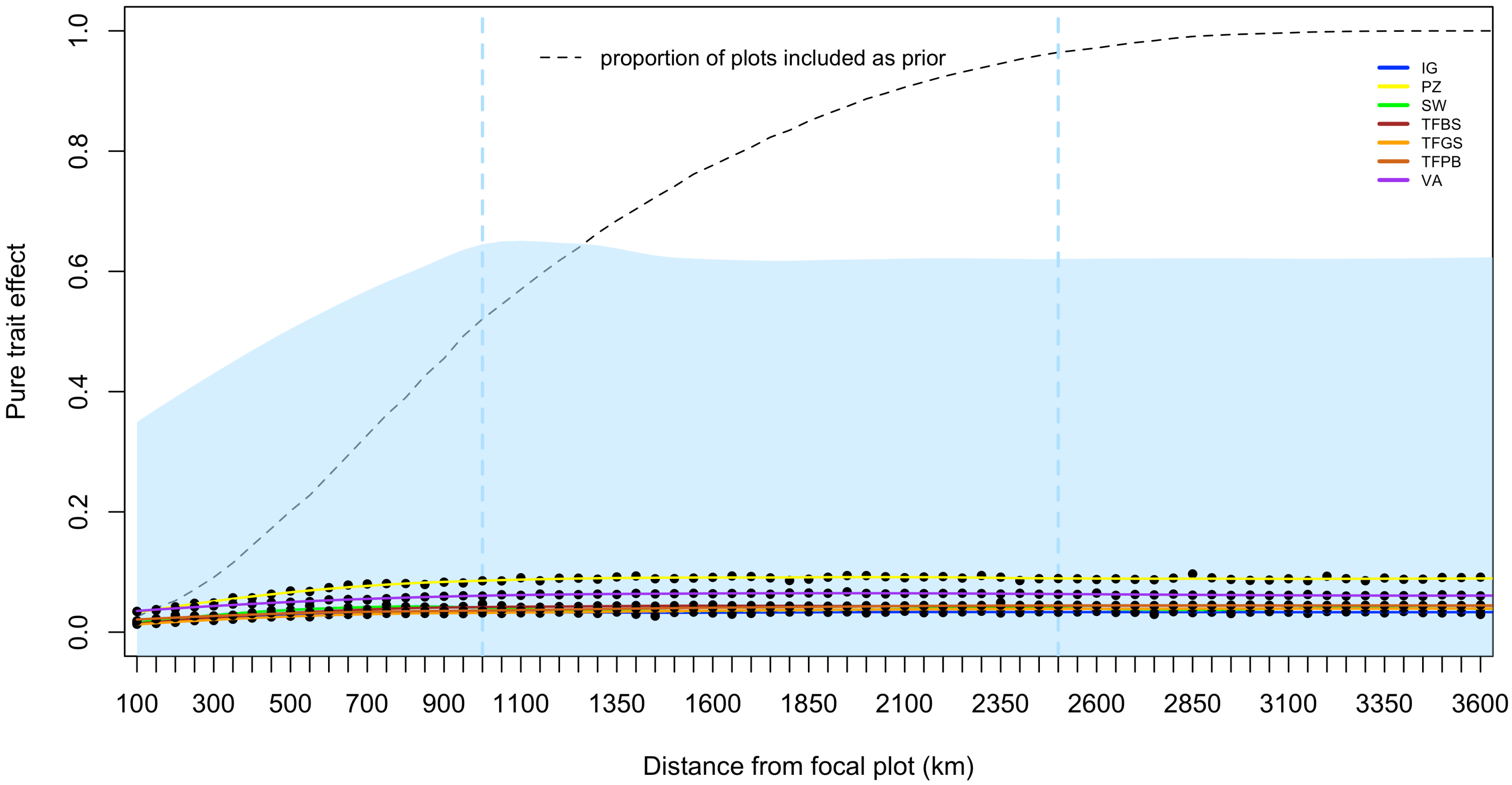

Fig. S4. Distance decay of pure trait effect for each forest type separately and the overall dataset. X-axis represents the radius of the metacommunity prior; i.e. the first 100 km consists of just a few plots and at 3800 km all plots are taken into account. Y-axis represent the pure trait effect, i.e. the increase in explained deviance due to traits beyond contributions made by the meta-community and relative to the model bias (see also Box S2). Colors indicate the different forest types with abbreviations as in main text. Lines indicate the predictions following from the loess regression based on all points. Blue vertical lines indicate the 1000 and 2500 km boundary points. Blue shading reflects maximum values for that distance of the whole dataset.

FIGURE S5

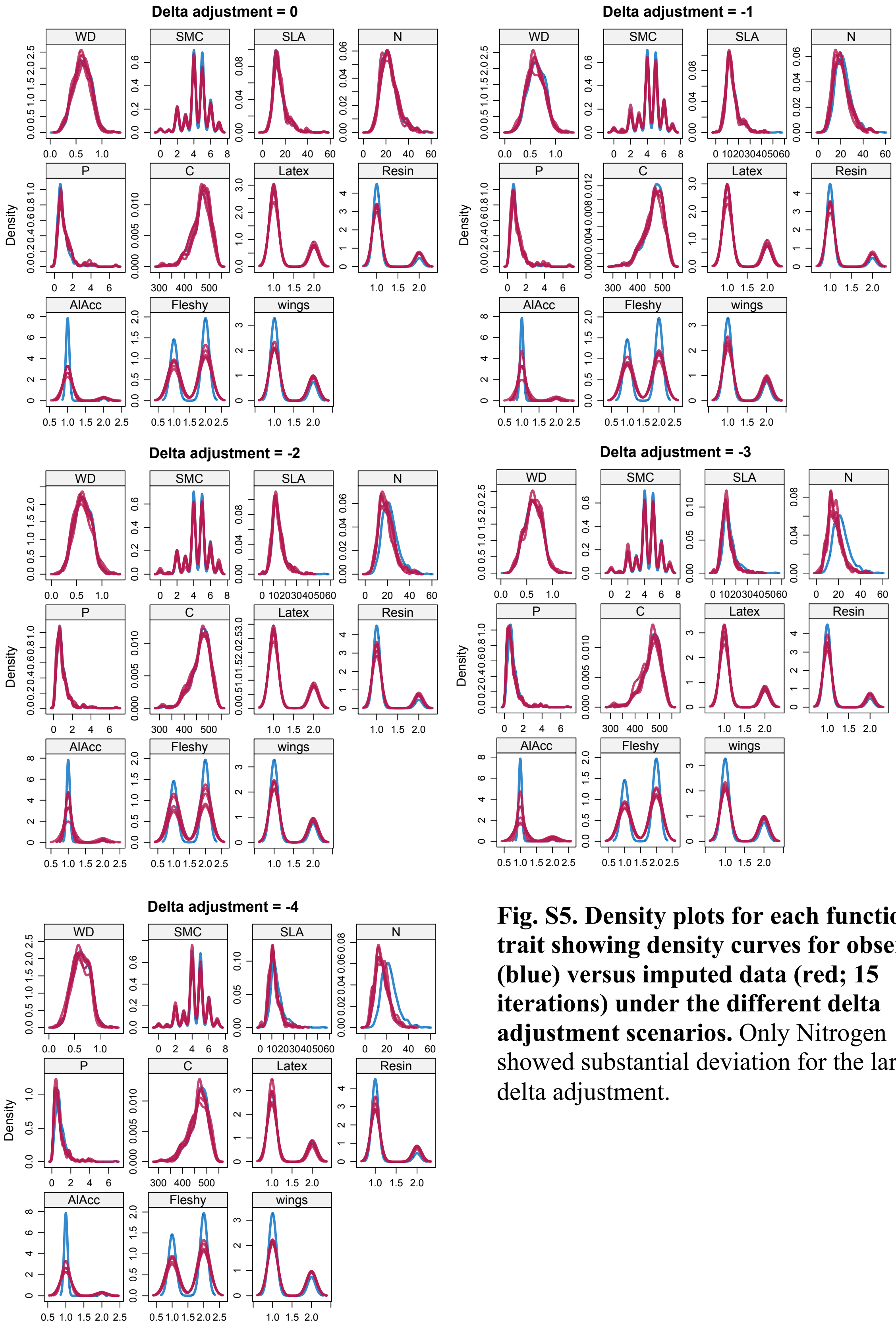

Fig. S5. Density plots for each functional trait showing density curves for observed (blue) versus imputed data (red; 15 iterations) under the different delta adjustment scenarios. Only Nitrogen showed substantial deviation for the larger delta adjustment.

FIGURE S6

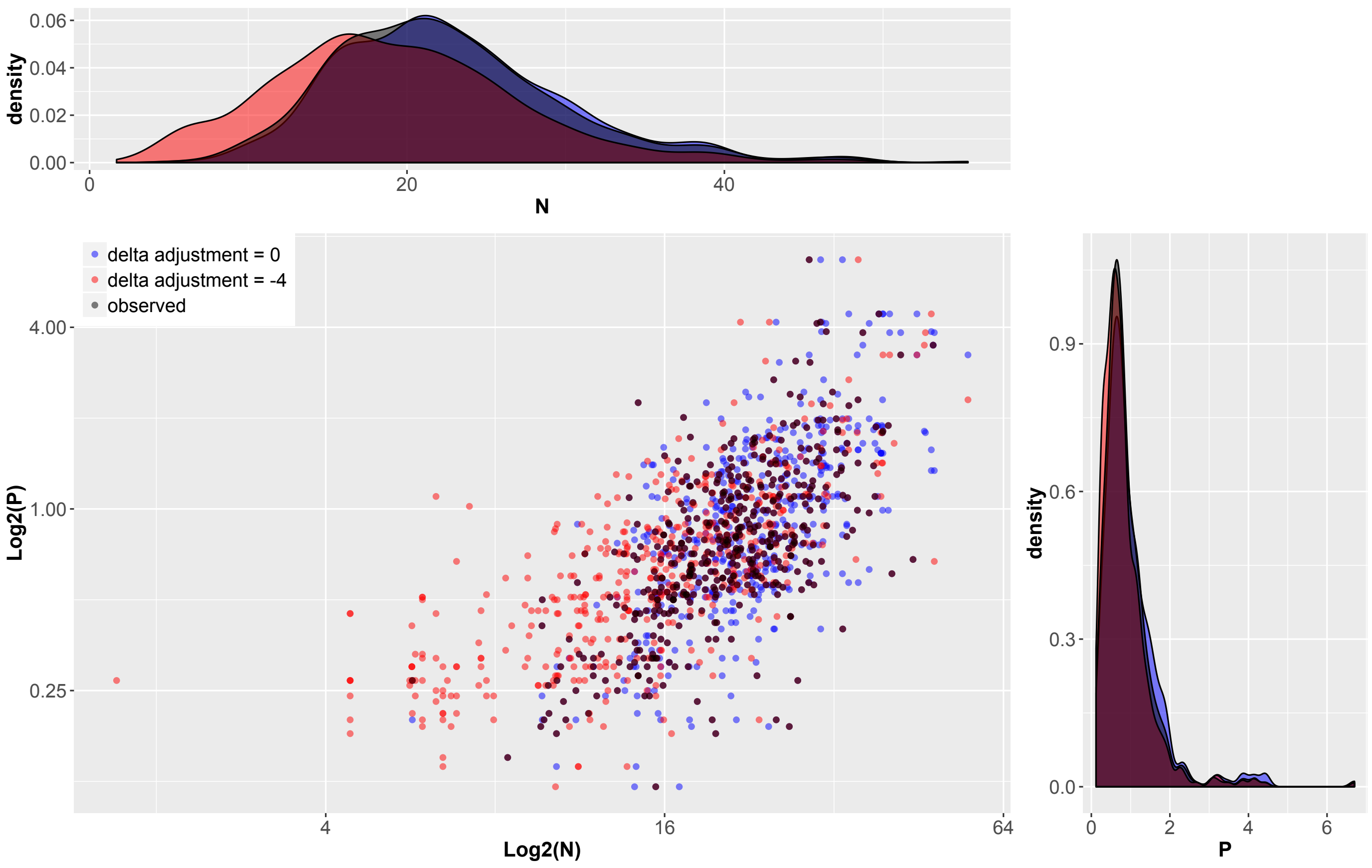

Fig. S6. Scatterplot of observed vs. imputed leaf nitrogen and phosphorus content under different delta adjustment scenarios. As Leaf Nitrogen content showed substantial deviation in the larger delta adjustment scenario it was here plotted versus Leaf Phosphorus content, which showed hardly any deviation.

FIGURE S7

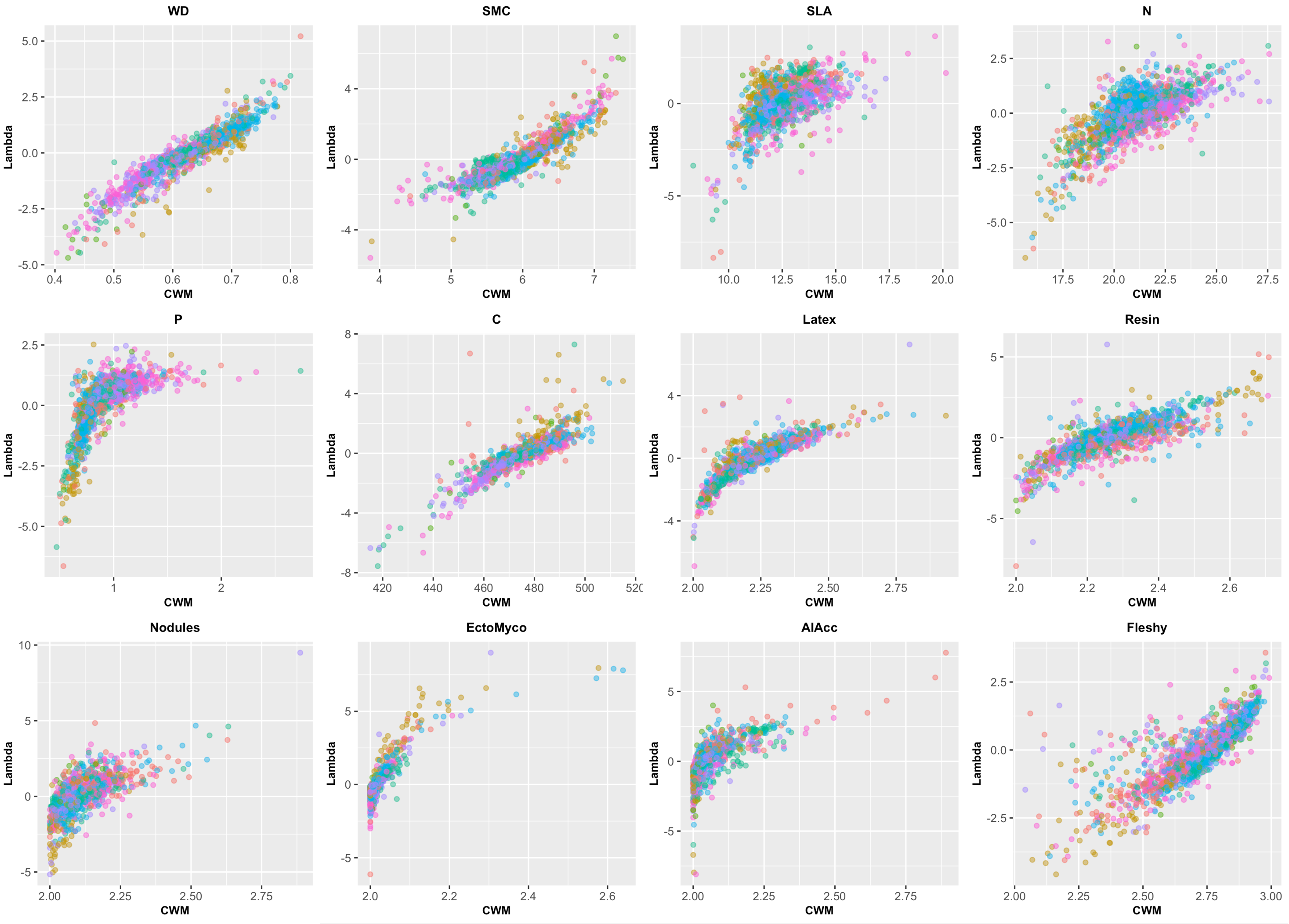

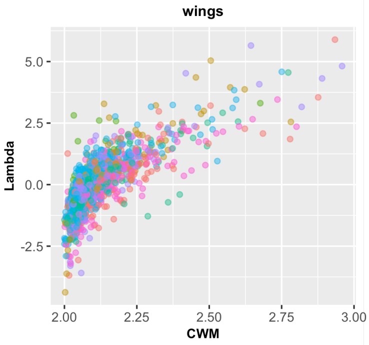

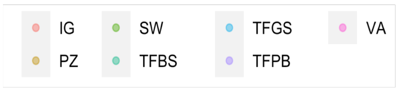

Fig. S7 Scatterplots of the CWM values against lambda values, colored according to forest type. Titles are abbreviations for functional traits as used in the main text. Plots show in some cases these are clearly correlated (e.g. wood density, C) but for many others not (e.g. SMC and AlAcc).

FIGURE S8

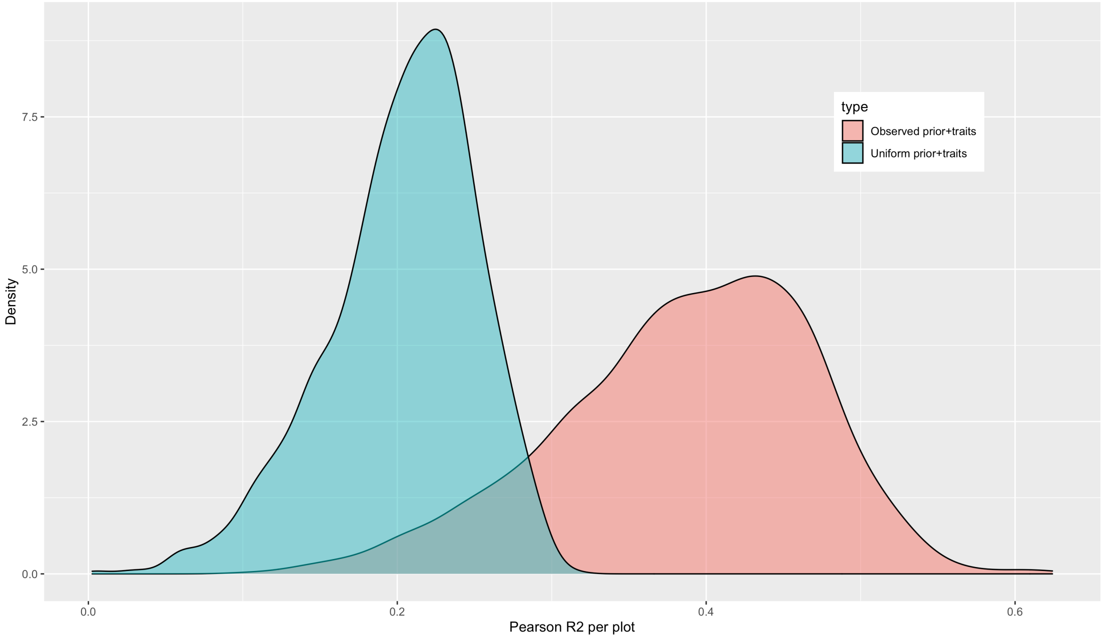

Fig. S8 Density plot of the per-plot-Pearson correlation coefficient between predicted relative abundances of each genus. Models either used a uniform prior and functional traits (blue) or the actual observed prior and functional traits (red), results show a large increase in accuracy for the latter.

TABLE S1

Table S1. Decomposition of results from the various maximum entropy models, combined and separated by forest type (PZ podzol, IG i*gapó*, VA *várzea*, SW Swamp, TF *terra firme* with subregions BS (Brazilian Shield), GS (Guyana Shield) and PB (Pebas formation). Top rows indicate estimated proportions (R^2_{KL}) of total information reflective of variation in local relative abundance explained for by the various models. Middle rows indicate the specific information gain from any one of the used models relative to the model bias. Bottom rows show the actual effects of traits, the metacommunity and the joint information relative to the model bias.

	**Forest types**							
**Explained proportions**	PZ	VA	IG	SW	TFBS	TFGS	TFPB	Combined
$\bar{R}^2_{KL}(u)$<br>*model bias fit*	0.17	0.11	0.12	0.19	0.08	0.08	0.06	0.09
$\bar{R}^2_{KL}(m)$<br>*pure neutral model fit*	0.51	0.51	0.52	0.55	0.55	0.62	0.55	0.56
$R^2_{KL}(u,t)$<br>*pure trait model fit*	0.34	0.25	0.23	0.34	0.21	0.24	0.20	0.23
$R^2_{KL}(m,t)$<br>*hybrid model fit*	0.59	0.57	0.55	0.57	0.59	0.66	0.59	0.60
**Increase in explained deviance**								
$\Lambda R^2_{KL}(m\vert\varphi)$<br>*metacommunity effect beyond model bias*	0.35	0.40	0.40	0.36	0.47	0.54	0.48	0.47
$\Lambda R^2_{KL}(t\vert\varphi)$<br>*trait effect beyond model bias*	0.17	0.14	0.11	0.15	0.13	0.16	0.14	0.14
$\Lambda R^2_{KL}(t\vert m)$<br>*trait effect beyond metacommunity effect*	0.07	0.05	0.03	0.02	0.04	0.04	0.04	0.04
$\Lambda R^2_{KL}(m\vert t)$<br>*metacommunity effect relative to trait effects*	0.25	0.32	0.32	0.23	0.38	0.42	0.39	0.37
$\Lambda R^2_{KL}(m+t)$<br>*joint effect of metacommunity and traits*	0.10	0.08	0.08	0.13	0.09	0.12	0.09	0.10
$1- \Lambda R^2_{KL}(m,t)$<br>*unexplained effects*	0.41	0.43	0.45	0.43	0.41	0.34	0.41	0.40
**Biologically relevant information**								
Pure trait effect<br>*Information from traits, relative to bias*	0.09	0.06	0.04	0.03	0.05	0.04	0.05	0.05
Pure metacommunity effect<br>*Information from metacommunity, relative to bias*	0.30	0.36	0.37	0.26	0.42	0.45	0.41	0.40
Joint effect<br>*Information from joint effect, relative to bias*	0.11	0.09	0.09	0.17	0.09	0.13	0.10	0.11
Unexplained information<br>*Left over information not explained, relative to bias*	0.49	0.49	0.51	0.54	0.44	0.38	0.44	0.44

TABLE S2

Table S2. Summary statistics overview for the linear models of the various scenarios under the delta adjustment technique as described in the main text. Rows indicate the different delta adjustments used with the columns representing the standard summary statistics of the linear model comparing the imputed versus observed trait values. Results showed similar patterns with each imputation scenario, indicating a robust imputation procedure.

	Summary statistics linear model lm(imputed ~ observed)						
**Scenario**	Intercept	Std. error	T value	Pr.	R^2	Adj. R^2	Sigma
Delta 0	-.33	.07	-4.85	1.42e-06	.32	.33	.63
Delta -2.5	-.34	.06	-5.93	4.31e-09	.37	.37	.58
Delta -5	-.16	.04	-3.57	3.77e-04	.40	.40	.53
Delta -7.5	.64	.09	-7.33	5.18e-13	.42	.42	.88
Delta -10	0.09	.04	3.17	1.57e-03	.47	.47	.48

S-A Ecological interpretation of the MEF results

Signals of quantitative environmental selection were found to be highest for podzol forests, whereas its counterpart in the form of the dispersal mass effect from the regional pool of genera had the second lowest value. Podzol forests, having extremely nutrient poor soils could reflect a much stronger selective environment than any of the other forest types. *Terra firme* forests, presumably reflective of a less strong selective environment in terms of resource availability, showed the opposite, with approximately half of the pure trait effect in comparison with podzol forests (even when rarefied to accommodate for different sample sizes). Traits associated with protection against herbivores such as latex [7] and high leaf carbon content showed higher values associated with greater abundance on podzol soils, whereas traits indicative of investment in growth and photosynthetic ability such as high foliar concentrations of P and N [8] showed strong negative associations on nutrient poor soils. The ability to accumulate aluminium was also strongly positively associated with relative abundance on *igapó* forests, which can potentially be richer in aluminium. Lambda values also showed strong negative lambda values for wood density in swamp and forests, fitting high tree mortality and many individuals belonging to pioneer species in especially the western Amazonian swamp forests. *Várzea* and Pebas *terra firme* forests showed a similar response. As the Pebas consists mainly of Andean sediments it has higher nutrient content, promoting lower wood density, supported by our results whereas *várzea* forests are also often flooded. There were also traits that showed no specific (strong) signal of selection on certain forest types (either positive or negative), such as latex on *igapó* and ectomycorrhiza on terra firme forests (see Fig. S1 for all lambda values). Interestingly, terra firme forests in general showed the smallest lambda values overall (positive or negative). This may be indicative of either more pronounced demographic stochasticity or ecological drift eliminating the association between traits and relative abundance. Lower effects of selection in general or more (random) variation due to the larger species pool in comparison with other forest types, however, could also be the result of mixing heterogeneous microenvironments into a single environmental class. Support for such heterogeneity within terra firme forests having influence on distribution of functional traits on valleys or plateaus has recently been found [9]. In addition, natural but also anthropogenic [10] disturbance history affects biotic community composition and can lead to changes in tree community through time, blurring relationships between traits and relative abundances. It should further be noted that, although for terra firme forests we were able to make a distinction by subregion, true within forest type heterogeneity was not taken into account. This might cause an underestimation of the deterministic effect but as of yet cannot be corrected for on this scale and is worth to be investigated in future studies. In addition, podzol forests have a smaller connected surface area and accompanying smaller number of genera in comparison with terra firme forests, adding to the calculated stronger trait effects [11,12]. When more detailed understanding and knowledge of these functional traits would be provided, this would most likely increase the explanatory power of the MEF. The fact, however, that we do not have a very specific knowledge of these interactions and specific traits is precisely the reason why the MEF can provide additional insight.

It should be noted that for species level analyses any micro environmental gradients might prove to also show (stronger) selection at local scales [13,14], as it has been shown that most variation in community composition, due to selection in regard to habitat filtering and niche conservatism, is found at lower taxonomic levels, such as between species within genera [15,16]. In contrast, theoretically it has been shown and tested that immigration numbers are actually very robust across taxonomic scales [17], validating our results of the metacommunity importance using genus level taxonomy. Spatial patterns of metacommunity effects showed shallowest declines in the centre, supporting the suggestion that high diversity of the Amazonian interior could be explained by influx of recruits due to large (overlapping) ranges. This mid-domain effect [18], however, would also predict lower species richness for the edges due to lower range overlap, assuming a closed community. This is not the case, as there is a strong species richness gradient from West (rich) to Eastern Amazonian forests (poor) [19]. The lower metacommunity effect for the edges then is most likely not due to less absolute influx of genera, but rather less influx from the Amazonian tree community. Influx from the species-rich Andes could account for the high diversity [20], yet low Amazonian metacommunity effect for Western Amazonian forests. In contrast, South-eastern parts of Amazonia receive influx from tree species-poor biomes (i.e. the Cerrado) resulting in lower diversity but also low metacommunity effect for Amazonian trees in this region.

S-A2 List of packages used in addition to standard R [1] preloaded packages

Vegan Jari Oksanen, F. Guillaume Blanchet, Michael Friendly, Roeland Kindt, Pierre Legendre, Dan McGlinn, Peter R. Minchin, R. B. O'Hara, Gavin L. Simpson, Peter Solymos, M. Henry H. Stevens, Eduard Szoecs and Helene Wagner (2020). vegan: Community Ecology Package. R package version 2.5-7. https://CRAN.R-project.org/package=vegan

FD Laliberté, E., and P. Legendre (2010) A distance-based framework for measuring functional diversity from multiple traits. Ecology 91:299-305.

FD Laliberté, E., Legendre, P., and B. Shipley. (2014). FD: measuring functional diversity from multiple traits, and other tools for functional ecology. R package version 1.0-12.

Fields Douglas Nychka, Reinhard Furrer, John Paige, Stephan Sain (2021). "fields: Tools for spatial data." R package version 13.3, https://github.com/dnychka/fieldsRPackage

Binr Sergei Izrailev (2015). binr: Cut Numeric Values into Evenly Distributed Groups. R package version 1.1. https://CRAN.R-project.org/package=binr

doParallel Microsoft Corporation and Steve Weston (2020). doParallel: Foreach Parallel Adaptor for the 'parallel' Package. R package version 1.0.16. https://CRAN.R-project.org/package=doParallel

Itertools Steve Weston and Hadley Wickham (2014). itertools: Iterator Tools. R package version 0.1-3. https://CRAN.R-project.org/package=itertools

Reshape Hadley Wickham (2007). Reshaping Data with the reshape Package. Journal of Statistical Software, 21(12), 1-20. URL http://www.jstatsoft.org/v21/i12/.

Ggplot2 H. Wickham. ggplot2: Elegant Graphics for Data Analysis. Springer-Verlag New York, 2016.

Plotrix Lemon, J. (2006) Plotrix: a package in the red light district of R. R-News, 6(4): 8-12.

Mice Stef van Buuren, Karin Groothuis-Oudshoorn (2011). mice: Multivariate Imputation by Chained Equations in R. Journal of Statistical Software, 45(3), 1-67. DOI 10.18637/jss.v045.i03.

REFERENCES SUPPLEMENTARY MATERIAL

[1] B. Shipley, *From Plant Traits to Vegetation Structure. Chance and Selection in the Assembly of Ecological Communities* (Cambridge University Press, 2010).

[2] B. Shipley, C. E. T. Paine, and C. Baraloto, *Quantifying the Importance of Local Niche-Based and Stochastic Processes to Tropical Tree Community Assembly*, Ecology **93**, 760 (2012).

[3] J. Loranger, F. Munoz, B. Shipley, and C. Violle, *What Makes Trait–Abundance Relationships When Both Environmental Filtering and Stochastic Neutral Dynamics Are at Play?*, Oikos **127**, 1735 (2018).

[4] A. Colin Cameron and F. A. G. Windmeijer, *An R-Squared Measure of Goodness of Fit for Some Common Nonlinear Regression Models*, J. Econom. **77**, 329 (1997).

[5] B. Shipley, *Measuring and Interpreting Trait-Based Selection versus Meta-Community Effects during Local Community Assembly*, J. Veg. Sci. **25**, 55 (2014).

[6] H. ter Steege, N. C. a Pitman, O. L. Phillips, J. Chave, D. Sabatier, A. Duque, J.-F. Molino, M.-F. Prévost, R. Spichiger, H. Castellanos, P. von Hildebrand, and R. Vásquez, *Continental-Scale Patterns of Canopy Tree Composition and Function across Amazonia.*, Nature **443**, 444 (2006).

[7] A. A. Agrawal, *Macroevolution of Plant Defense Strategies*, Trends Ecol. Evol. **22**, 103 (2007).

[8] J. C. Ordoñez, P. M. Van Bodegom, J. P. M. Witte, I. J. Wright, P. B. Reich, and R. Aerts, *A Global Study of Relationships between Leaf Traits, Climate and Soil Measures of Nutrient Fertility*, Glob. Ecol. Biogeogr. **18**, 137 (2009).

[9] R. S. Oliveira, F. R. C. Costa, E. van Baalen, A. de Jonge, P. R. Bittencourt, Y. Almanza, F. de V. Barros, E. C. Cordoba, M. V. Fagundes, S. Garcia, Z. T. T. M. Guimaraes, M. Hertel, J. Schietti, J. Rodrigues-Souza, and L. Poorter, *Embolism Resistance Drives the Distribution of Amazonian Rainforest Tree Species along Hydro-Topographic Gradients*, New Phytol. (2018).

[10] C. Levis, B. M. Flores, P. A. Moreira, B. G. Luize, R. P. Alves, J. Franco-Moraes, J. Lins, E. Konings, M. Peña-Claros, F. Bongers, F. R. C. Costa, and C. R. Clement, *How People Domesticated Amazonian Forests*, Front. Ecol. Evol. **5**, (2018).

[11] J. E. Guevara, G. Damasco, C. Baraloto, P. V. A. Fine, M. C. Peñuela, C. Castilho, A. Vincentini, D. Cárdenas, F. Wittmann, N. Targhetta, O. Phillips, J. Stropp, I. Amaral, P. Maas, A. Monteagudo, E. M. Jimenez, R. Thomas, R. Brienen, Á. Duque, W. Magnusson, C. Ferreira, E. Honorio, F. de Almeida Matos, F. R. Arevalo, J. Engel, P. Petronelli, R. Vasquez, and H. ter Steege, *Low Phylogenetic Beta Diversity and Geographic Neo-Endemism in Amazonian White-Sand Forests*, Biotropica **48**, 34 (2016).

[12] H. ter Steege, N. C. A. Pitman, D. Sabatier, C. Baraloto, R. P. Salomão, J. E. Guevara, O. L. Phillips, C. V Castilho, W. E. Magnusson, J.-F. Molino, A. Monteagudo, P. Núñez Vargas, J. C. Montero, T. R. Feldpausch, E. N. H. Coronado, T. J. Killeen, B. Mostacedo, R. Vasquez, R. L. Assis, J. Terborgh, F. Wittmann, A. Andrade, W. F. Laurance, S. G. W. Laurance, B. S. Marimon, B.-H. Marimon, I. C. Guimarães Vieira, I. L. Amaral, R. Brienen, H. Castellanos, D. Cárdenas López, J. F. Duivenvoorden, H. F. Mogollón, F. D. de A. Matos, N. Dávila, R. García-Villacorta, P. R. Stevenson Diaz, F. Costa, T. Emilio, C. Levis, J. Schietti, P. Souza, A. Alonso, F. Dallmeier, A. J. D. Montoya, M. T. Fernandez Piedade, A. Araujo-Murakami, L. Arroyo, R. Gribel, P. V. A. Fine, C. A. Peres, M. Toledo, G. A. Aymard C, T. R. Baker, C. Cerón, J. Engel, T. W. Henkel, P. Maas, P. Petronelli, J. Stropp, C. E. Zartman, D. Daly, D. Neill, M. Silveira, M. R. Paredes, J. Chave, D. de A. Lima Filho, P. M. Jørgensen, A. Fuentes, J. Schöngart, F. Cornejo Valverde, A. Di Fiore, E. M. Jimenez, M. C. Peñuela Mora, J. F. Phillips, G. Rivas, T. R. van Andel, P. von Hildebrand, B. Hoffman, E. L. Zent, Y. Malhi, A. Prieto, A. Rudas, A. R. Ruschell, N. Silva, V. Vos, S. Zent, A. A. Oliveira, A. C. Schutz, T. Gonzales, M. Trindade Nascimento, H. Ramirez-Angulo, R. Sierra, M. Tirado, M. N. Umaña Medina, G. van der Heijden, C. I. A. Vela, E. Vilanova Torre, C. Vriesendorp, O. Wang, K. R. Young, C. Baider, H. Balslev, C. Ferreira, I. Mesones, A. Torres-Lezama, L. E. Urrego Giraldo, R. Zagt, M. N. Alexiades, L. Hernandez, I. Huamantupa-Chuquimaco, W. Milliken, W. Palacios Cuenca, D. Pauletto, E. Valderrama Sandoval, L. Valenzuela Gamarra, K. G. Dexter, K. Feeley, G. Lopez-Gonzalez, M. R. Silman, S. P. Hubbell, F. He, R. Condit, L. Borda-de-Agua, J. Kellner, H. Ter Steege, G. A. Black, T. H. Dobzhansky, C. Pavan, J. M. Pires, T. Dobzhansky, G. A. Black, M. J. G. Hopkins, M. J. Costello, R. M. May, N. E. Stork, P. Haripersaud, H. ter Steege, J.-J. de Granville, H. Chevillotte, M. Hoff, D. P. Bebber, M. A. Carine, J. R. Wood, A. H. Wortley, D. J. Harris, G. T. Prance, G. Davidse, J. Paige, T. D. Pennington, N. K. Robson, R. W. Scotland, B. J. McGill, R. S. Etienne, J. S. Gray, D. Alonso, M. J. Anderson, H. K. Benecha, M. Dornelas, B. J. Enquist, J. L. Green, F. He, A. H. Hurlbert, A. E. Magurran, P. A. Marquet, B. A. Maurer, A. Ostling, C. U. Soykan, K. I. Ugland, E. P. White, R. J. Warren, D. K. Skelly, O. J. Schmitz, M. A. Bradford, N. C. A. Pitman, J. W. Terborgh, M. R. Silman, P. N. V, D. A. Neill, C. E. Cerón, W. A. Palacios, M. Aulestia, N. C. A. Pitman, M. R. Silman, J. W. Terborgh, F. D. Lozano, M. W. Schwartz, M. W. Schwartz, D. Simberloff, J. E. Richardson, R. T. Pennington, T. D. Pennington, P. M. Hollingsworth, T. L. Couvreur, F. Forest, W. J. Baker, S. Cavers, C. W. Dick, D. H. Janzen, S. A. Mangan, S. A. Schnitzer, E. A. Herre, K. M. Mack, M. C. Valencia, E. I. Sanchez, J. D. Bever, W. Balée, D. G. Campbell, C. Levis, P. F. de Souza, J. Schietti, T. Emilio, J. L. P. V. Pinto, C. R. Clement, F. R. C. Costa, D. A. Posey, E. Montoya, V. Rull, N. D. Stansell, M. B. Abbott, S. Nogué, B. W. Bird, W. A. Díaz, C. Gomez-Navarro, C. Jaramillo, F. Herrera, S. L. Wing, R. Callejas, C. H. McMichael, D. R. Piperno, M. B. Bush, M. R. Silman, A. R. Zimmerman, M. F. Raczka, L. C. Lobato, H. ter Steege, P. P. Haripersaud, O. S. Bánki, F. Schieving, S. J. Phillips, R. P. Anderson, R. E. Schapire, S. J. Phillips, M. Dudik, C. A. Quesada, J. Lloyd, L. O. Anderson, N. M. Fyllas, M. Schwarz, C. I. Czimczik, B. Rollet, H. ter Steege, N. C. Pitman, O. L. Phillips, J. Chave, D. Sabatier, A. Duque, J. F. Molino, M. F. Prévost, R. Spichiger, H. Castellanos, P. von Hildebrand, R. Vásquez, P. M. Fearnside, D.

Mouillot, D. R. Bellwood, C. Baraloto, J. Chave, R. Galzin, M. Harmelin-Vivien, M. Kulbicki, S. Lavergne, S. Lavorel, N. Mouquet, C. E. Paine, J. Renaud, W. Thuiller, G. Lopez-Gonzalez, S. L. Lewis, M. Burkitt, O. L. Phillips, P. J. M. Maas, L. Y. T. Westra, H. Rainer, A. Q. Lobão, R. H. J. Erkens, P. V. A. Fine, D. C. Daly, G. V. Muñoz, I. Mesones, K. M. Cameron, K. J. Feeley, M. R. Silman, M. Dufrene, P. Legendre, J. Bunge, M. Fitzpatrick, J. Bunge, L. Woodard, D. Böhning, J. A. Foster, S. Connolly, H. K. Allen, J.-P. Z. Wang, B. G. Lindsay, A. Chao, R. K. Colwell, C.-W. Lin, N. J. Gotelli, C. X. Mao, R. K. Colwell, U. Brose, N. D. Martinez, R. J. Williams, I. J. Good, A. Chao, S.-M. Lee, I. Rocchetti, J. Bunge, D. Bohning, R. A. Fisher, A. S. Corbet, C. B. Williams, F. W. Preston, J.-P. Wang, P. V. A. Fine, and R. H. Ree, *Hyperdominance in the Amazonian Tree Flora*, Science **342**, 1243092 (2013).

[13] L. H. M. Cosme, J. Schietti, F. R. C. Costa, and R. S. Oliveira, *The Importance of Hydraulic Architecture to the Distribution Patterns of Trees in a Central Amazonian Forest*, New Phytol. **215**, 113 (2017).

[14] S. E. Russo, S. J. Davies, D. A. King, and S. Tan, *Soil-Related Performance Variation and Distributions of Tree Species in a Bornean Rain Forest*, J. Ecol. **93**, 879 (2005).

[15] K. J. Gaston, *Species-Range Size Distributions: Products of Speciation, Extinction and Transformation: Philosophical Transactions of the Royal Society of London Series B*, **353**, 219 (1998).

[16] R. E. Ricklefs, H. Qian, and P. S. White, *The Region Effect on Mesoscale Plant Species Richness between Eastern Asia and Eastern North America*, **2**, 129 (2004).

[17] F. Munoz, B. R. Ramesh, and P. Couteron, *How Do Habitat Filtering and Niche Conservatism Affect Community Composition at Different Taxonomic Resolutions?*, Ecology **95**, 2179 (2014).

[18] R. K. Colwell, C. Rahbek, and N. J. Gotelli, *The Mid-Domain Effect and Species Richness Patterns:What Have We Learned so Far?*, Am. Nat. **163**, E1 (2004).

[19] H. ter Steege, N. Pitman, D. Sabatier, H. Castellanos, P. Van der Hout, D. C. Daly, M. Silveira, O. Phillips, R. Thomas, J. V. A. N. Essen, H. Mogollon, and W. Morawetz, *A Spatial Model of Tree a -Diversity and Tree Density for the Amazon*, Biodivers. Conserv. **12**, 2255 (2003).

[20] T. F. Rangel, N. R. Edwards, P. B. Holden, J. A. F. Diniz-Filho, W. D. Gosling, M. T. P. Coelho, F. A. S. Cassemiro, C. Rahbek, and R. K. Colwell, *Modeling the Ecology and Evolution of Biodiversity: Biogeographical Cradles, Museums, and Graves. Science*, Science. **361**, (2018).
